# A Human Type 5 Adenovirus-Based *Trypanosoma cruzi* Therapeutic Vaccine Re-programs Immune Response and Reverses Chronic Cardiomyopathy

**DOI:** 10.1371/journal.ppat.1004594

**Published:** 2015-01-24

**Authors:** Isabela Resende Pereira, Glaucia Vilar-Pereira, Virgínia Marques, Andrea Alice da Silva, Bráulia Caetano, Otacilio Cruz Moreira, Alexandre Vieira Machado, Oscar Bruna-Romero, Maurício Martins Rodrigues, Ricardo Tostes Gazzinelli, Joseli Lannes-Vieira

**Affiliations:** 1 Laboratório de Biologia das Interações, Instituto Oswaldo Cruz/Fiocruz, Rio de Janeiro, Rio de Janeiro, Brazil; 2 Departamento de Patologia, Universidade Federal Fluminense, Niterói, Rio de Janeiro, Brazil; 3 Centro de Pesquisas René Rachou, Fiocruz, Belo Horizonte, Minas Gerais, Brazil; 4 Departamento de Bioquímica e Imunologia, Instituto de Ciências Biológicas, Universidade Federal de Minas Gerais, Belo Horizonte, Minas Gerais, Brazil; 5 Laboratório de Vírus Respiratórios e do Sarampo, Instituto Oswaldo Cruz/Fiocruz, Rio de Janeiro, Rio de Janeiro, Brazil; 6 Laboratório de Biologia Molecular e Doenças Endêmicas, Instituto Oswaldo Cruz/Fiocruz, Rio de Janeiro, Rio de Janeiro, Brazil; 7 Departamento de Microbiologia, Imunologia e Parasitologia, Universidade Federal de Santa Catarina, Florianopolis, Santa Catarina, Brazil; 8 Departamento de Microbiologia, Imunologia e Parasitologia, Universidade Federal de São Paulo-Escola Paulista de Medicina, São Paulo, São Paulo, Brazil; 9 Division of Infectious Disease and Immunology, Department of Medicine, University of Massachusetts Medical School, Worcester, Massachusetts, United States of America; University of Texas Medical Branch, UNITED STATES

## Abstract

Chagas disease (CD), caused by the protozoan *Trypanosoma cruzi*, is a prototypical neglected tropical disease. Specific immunity promotes acute phase survival. Nevertheless, one-third of CD patients develop chronic chagasic cardiomyopathy (CCC) associated with parasite persistence and immunological unbalance. Currently, the therapeutic management of patients only mitigates CCC symptoms. Therefore, a vaccine arises as an alternative to stimulate protective immunity and thereby prevent, delay progression and even reverse CCC. We examined this hypothesis by vaccinating mice with replication-defective human Type 5 recombinant adenoviruses (rAd) carrying sequences of amastigote surface protein-2 (rAdASP2) and trans-sialidase (rAdTS) *T. cruzi* antigens. For prophylactic vaccination, naïve C57BL/6 mice were immunized with rAdASP2+rAdTS (rAdVax) using a homologous prime/boost protocol before challenge with the Colombian strain. For therapeutic vaccination, rAdVax administration was initiated at 120 days post-infection (dpi), when mice were afflicted by CCC. Mice were analyzed for electrical abnormalities, immune response and cardiac parasitism and tissue damage. Prophylactic immunization with rAdVax induced antibodies and H-2K^b^-restricted cytotoxic and interferon (IFN)γ-producing CD8^+^ T-cells, reduced acute heart parasitism and electrical abnormalities in the chronic phase. Therapeutic vaccination increased survival and reduced electrical abnormalities after the prime (analysis at 160 dpi) and the boost (analysis at 180 and 230 dpi). Post-therapy mice exhibited less heart injury and electrical abnormalities compared with pre-therapy mice. rAdVax therapeutic vaccination preserved specific IFNγ-mediated immunity but reduced the response to polyclonal stimuli (anti-CD3 plus anti-CD28), CD107a^+^ CD8^+^ T-cell frequency and plasma nitric oxide (NO) levels. Moreover, therapeutic rAdVax reshaped immunity in the heart tissue as reduced the number of perforin+ cells, preserved the number of IFNγ^+^ cells, increased the expression of IFNγ mRNA but reduced inducible NO synthase mRNA. Vaccine-based immunostimulation with rAd might offer a rational alternative for re-programming the immune response to preserve and, moreover, recover tissue injury in Chagas’ heart disease.

## Author Summary

The idea that Chagas disease (CD) has an important autoimmune involvement contributed to delay the development of therapies and vaccines. CD is a parasitic neglected disease which afflicts millions of people mostly in Latin America. The cardiac form is the main clinical manifestation of CD. Currently, patients with access to therapy receive medicaments that only mitigate symptoms. Because of the limited prospect of treatment, vaccine reemerged as a strategy to prevent infection, interfere with CD progression and, moreover, reverse heart abnormalities. Here we tested a recombinant adenovirus carrying sequences of ASP2 and TS *T. cruzi* antigens (rAdVax) as prophylactic and therapeutic tool using a model of chronic Chagas’ heart disease. We showed that prophylactic vaccination reduced heart parasite load, inflammation and electrical abnormalities. The rAdVax therapeutic vaccination also reduced heart injury and improved electrical function, preserved specific IFNγ-mediated immunity but reduced response to polyclonal stimuli, CD107a^+^ CD8^+^ T-cell frequency and plasma nitric oxide levels. Moreover, therapeutic rAdVax preserved the number IFNγ^+^ cells, but decreased perforin^+^ cells in the heart tissue. Therefore, our results support the hypothesis that vaccination can modify the immunological unbalance that concurs to Chagas’ heart disease to improve prognosis.

## Introduction

Chagas disease (CD) is a neglected tropical illness caused by the protozoan parasite *Trypanosoma cruzi*, which is transmitted by blood-sucking triatomines. The disease afflicts 8 to 15 million people in Latin America; more than 40,000 new cases occur every year and the rate of congenital transmission is greater than 14,000 cases per year. Furthermore, approximately 1 million immigrants to the USA and Europe have CD [1]. Despite the successful control of the main vector, an overview of the current challenges reveals the need for (i) permanent vector surveillance and attention to domestic and peri-domestic reservoirs of the parasite, (ii) new strategies to prevent or abrogate infection and (iii) new therapies for patients with chronic forms of CD [2].

The most frequent and severe manifestation of CD is chronic chagasic cardiomyopathy (CCC), which is associated with inflammation, myocytolysis and fibrosis and affects 20–40% of infected individuals at 10–30 years after infection [1]. Innate and adaptive immunity play pivotal roles in parasite growth control during the acute phase of infection, allowing the establishment of chronic phase [3]. However, in patients with Chagas’ heart disease the natural immune response is mostly inadequate as parasite persistence and parasite-induced deregulated immune response are consensual explanations for CCC pathogenesis [4, 5]. Several studies have proposed veterinary vaccine as tools to prevent infection, particularly to decrease parasitemia in hosts and reservoirs, such as dogs, to control the domestic and peri-domestic cycle [6]. Furthermore, human vaccines would generate a positive return on investment, because such vaccines would prevent the onset of CCC and provide both cost savings and health benefits [7]. In chronic *T. cruzi* infections, vaccination should be considered as a therapeutic strategy to redirect immunity to a protective status to delay disease progression and reverse heart alterations in chronic patients.

Many attempts to generate a prophylactic vaccine for CD have been conducted in the last three decades, including use of the attenuated parasite, purified protein, recombinant protein and DNA and, more recently, replication-deficient bacteria and recombinant viral vectors to reduce acute parasitism and heart inflammation [8–11] and chronic myocarditis [12].

The amastigote surface protein-2 (ASP2), a protein with unknown function [13] and *trans*-sialidase (TS), a trypomastigote-restricted enzyme that catalyzes the transfer of sialic acid from host glycoproteins to acceptor molecules on the parasite membrane [14], are two of the most promising candidates for vaccine development. Researchers have attempted to produce an immunotherapeutic vaccine; however, these preparations were unable to control disease progression [10]. Furthermore, no vaccines that can reverse chronic Chagas’ heart disease are available.

Vaccines using replication-deficient human recombinant Type 5 adenoviruses (AdHu5) carrying sequences of the ASP2 (rAdASP2) and TS (rAdTS) proteins of the Y *T. cruzi* Type II strain [15] elicited Th1-biased immunity with a substantial CD8^+^ T-cell-mediated long-term protective immune response against challenge with the Y strain [16]. Furthermore, heterologous priming with plasmid DNA and boosting with rAdASP2 and rAdTS protected mice from challenges with the CL and Colombian *T. cruzi* strains, thereby demonstrating cross protective immunity [17]. Based on the results of previous studies, we tested the therapeutic properties of combined rAdASP2+rAdTS (rAdVax) in a homologous prime-boost protocol to skew the immune response to prevent, hamper progression and potentially reverse chronic Chagas’ heart disease. Therefore, we challenged the idea that unappropriated immune response contributes to cardiac abnormalities and once it has been reshaped heart damage progression may be delayed and, even, reversed.

## Results

### Prophylactic homologous prime-boost rAdVax administration reduces parasite burden and protects from electrical abnormalities in chronic experimental Chagas’ heart disease

Initially, we determined the ability of rAdVax to induce antibodies and specific CD8^+^ T-cells, which are considered protective in *T. cruzi* infection [16, 17]. To this end, C57BL/6 mice were vaccinated twice with rAdVax or rAdCtrl as described in the Materials and Methods section. Three to four weeks after boosting, the sera were collected and analyzed using ELISA and the CD8^+^ T-cell response was analyzed by in vivo cytotoxic assay and ELISpot for IFNγ detection (S1A Fig.). Specific total antibodies (IgM+IgG) against recombinant ASP2 and TS proteins were detected in rAdVax-immunized mice, whereas saline-injected or rAdCtrl-immunized mice presented negligible reactivity to these proteins (S1B Fig.). Protection against *T. cruzi* depends on CD8^+^ T-cell effector activities [4]. Significantly, high frequency of H-2k^b^-restricted anti-VNHRFTLV CTL CD8^+^ T-cells were detected in the spleen of rAdVax vaccinated mice but not in saline-injected or in rAdCtrl-immunized mice (S1C and S1D Fig.). Moreover, immunization with rAdVax induced a significant increase in the number of ASP2-specific IFNγ-producing CD8^+^ T-cells, which are of pivotal importance in *T. cruzi* growth control [3]. In contrast, specific IFNγ-producing CD8^+^ T-cells were absent in saline-injected mice and the number of these cells was reduced in rAdCtrl-immunized mice (S1E Fig.). Therefore, vaccination with rAdVax in a homologous prime-boost protocol stimulated anti-*T. cruzi* IgM+IgG antibodies and parasite-specific CTL and IFNγ-producing CD8^+^ T-cells, corroborating our previous data [16, 17].

**Figure 1:**
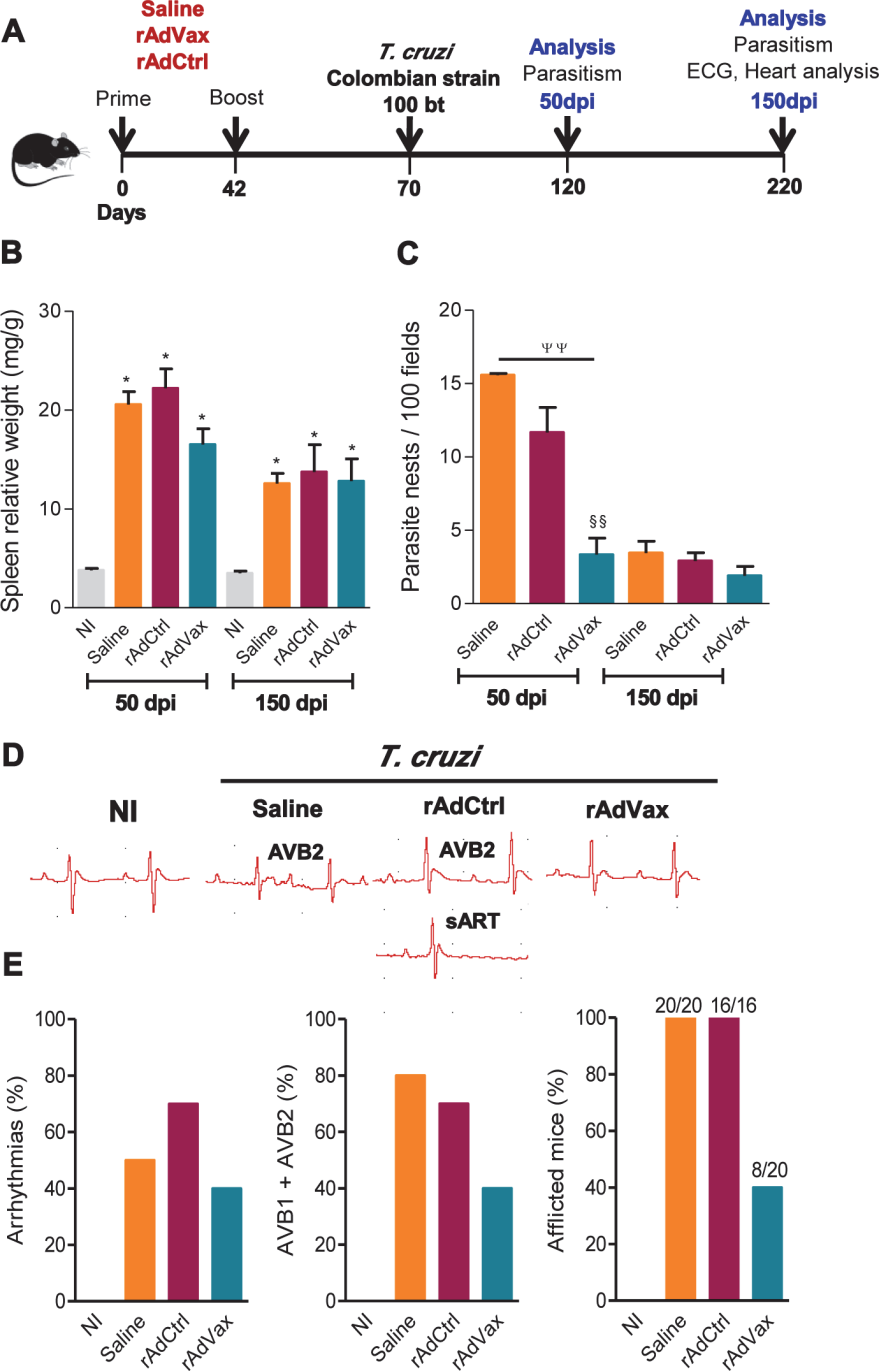
Heart parasitism and electrical abnormalities are reduced in rAdVax-vaccinated mice challenged with the Colombian strain. (**A**) Mice were primed-boosted (s.c.) with 2 × 10^8^ plaque-forming units (PFU) of rAdCtrl or a mixture of 10^8^ PFU of each recombinant adenovirus vaccine construction (rAdASP2+rAdTS; rAdVax) or the vehicle control saline at 6-week intervals. Four weeks after the last immunization, the mice were challenged (i.p.) with 100 blood trypomastigotes (bt) of the Colombian *T. cruzi* Type I strain and analyzed during the acute (50 dpi) and chronic (150 dpi) phases of infection. (**B**) Relative spleen weight (mg of spleen/g of body). (**C**) Quantitative immunohistochemical staining data for *T. cruzi* parasitism (nests/100 microscopic fields) in the heart tissue. (**D**) Representative electrocardiogram (ECG) register segments of sex- and age-matched noninfected (NI) controls and mice injected with saline, rAdCtrl or rAdVax, challenged with *T. cruzi* and analyzed at 150 dpi. (**E**) Summary of the group data from NI controls and saline-injected, rAdCtrl- or rAdVax-immunized and *T. cruzi*-infected mice showing the frequency of mice presenting arrhythmias (ART), second-degree atrioventricular block (AVB2) and afflicted with ECG alterations, at 150 dpi. The data are presented as the means ± SD of seven to ten mice per group. *P <0.05, experimental groups compared with NI controls. ^ΨΨ^ *P* < 0.01, rAdVax-immunized compared with saline-injected *T. cruzi*-infected mice. ^§§^ *P* < 0.01, rAdVax-immunized compared with rAdCtrl-injected *T. cruzi*-infected mice.

Initially, we tested whether prophylactic rAdVax administration could reduce heart parasitism and tissue damage in a model of acute phase of infection [17]. rAdVAx reduced the acute heart parasitism and cardiomyocyte damage induced by the CL-Brener *T. cruzi* Type VI strain (S2 Fig.). Next, we examined whether a homologous prime-boost vaccination with rAdVax could prevent *T. cruzi*-induced CCC. When C57BL/6 mice are infected with 100 blood trypomastigote (bt) forms of the Colombian strain, parasitemia peak occurs at 42–45 dpi, parasitemia control occurred from 60–70 dpi and chronic phase is established after 90 dpi, when none or rare blood trypomastigotes are detected [5]. Importantly, in this model of *T. cruzi* infection parasitemia and heart parasitism are directly associated and 70–85% of infected mice survived and developed a chronic disease with electrical abnormalities [5, 18]. Therefore, vaccinated C57BL/6 mice were challenged with 100 bt forms of the Colombian strain and tested for heart tissue parasitism and electrical alterations in the acute and chronic infection (Fig. 1A). The rAdVax vaccination did not alter parasitemia curve or the number of circulating parasites at the peak of parasitemia at 42 dpi (34.7 ± 17 × 10^3^ trypomastigotes/mL in rAdCtrl *vs*. 30.2 ± 15.7 × 10^3^ trypomastigotes/mL in rAdVax; *P* > 0.05). Although the prophylactic administration of rAdVax did not affect *T. cruzi*-induced splenomegaly (Fig. 1B), the number of parasite nests in the heart tissue was significantly reduced during the acute phase (Fig. 1C). rAdVax did not alter the numbers of CD4^+^ cells and F4/80^+^ macrophages, but reduced the number of CD8^+^ cells, infiltrating the cardiac tissue, at 50 dpi (S3 Fig.). Further, in rAdVax-vaccinated mice no significant alterations in myocarditis were detected at 150 dpi (2206 ± 719 inflammatory cell/100 microscopic fields in rAdCtrl *vs*. 1989 ± 934 inflammatory cell/100 microscopic fields in rAdVax; *P* > 0.05). Electrical abnormalities, including low heart rate, arrhythmia (ART) and first- and second-degree atrioventricular block (AVB1 and AVB2), are important features of the chronic cardiomyopathy induced by infection with the Colombian *T. cruzi* strain in C57BL/6 mice [5]. Notably, immunization with rAdVax remarkably decreased the frequency of mice presenting ART, particularly sinus arrhythmia (sART) and AVB2 at 150 dpi (Fig. 1D). Moreover, compared with rAdCtrl injection, rAdVax inoculation reduced the frequency of mice afflicted with ART, AVB1 (100% in saline-injected, 100% in rAdCtrl-treated and 20% in rAdVax), AVB2 and other electrical abnormalities (Fig. 1E). These observations supported our hypothesis that rAdVax is a feasible tool to ameliorate the outcome of chronic Chagas’ heart disease.

### Homologous prime-boost rAdVax immunotherapy during chronic infection delays progression and reverses chronic cardiomyopathy induced by the Colombian strain

**Figure 2:**
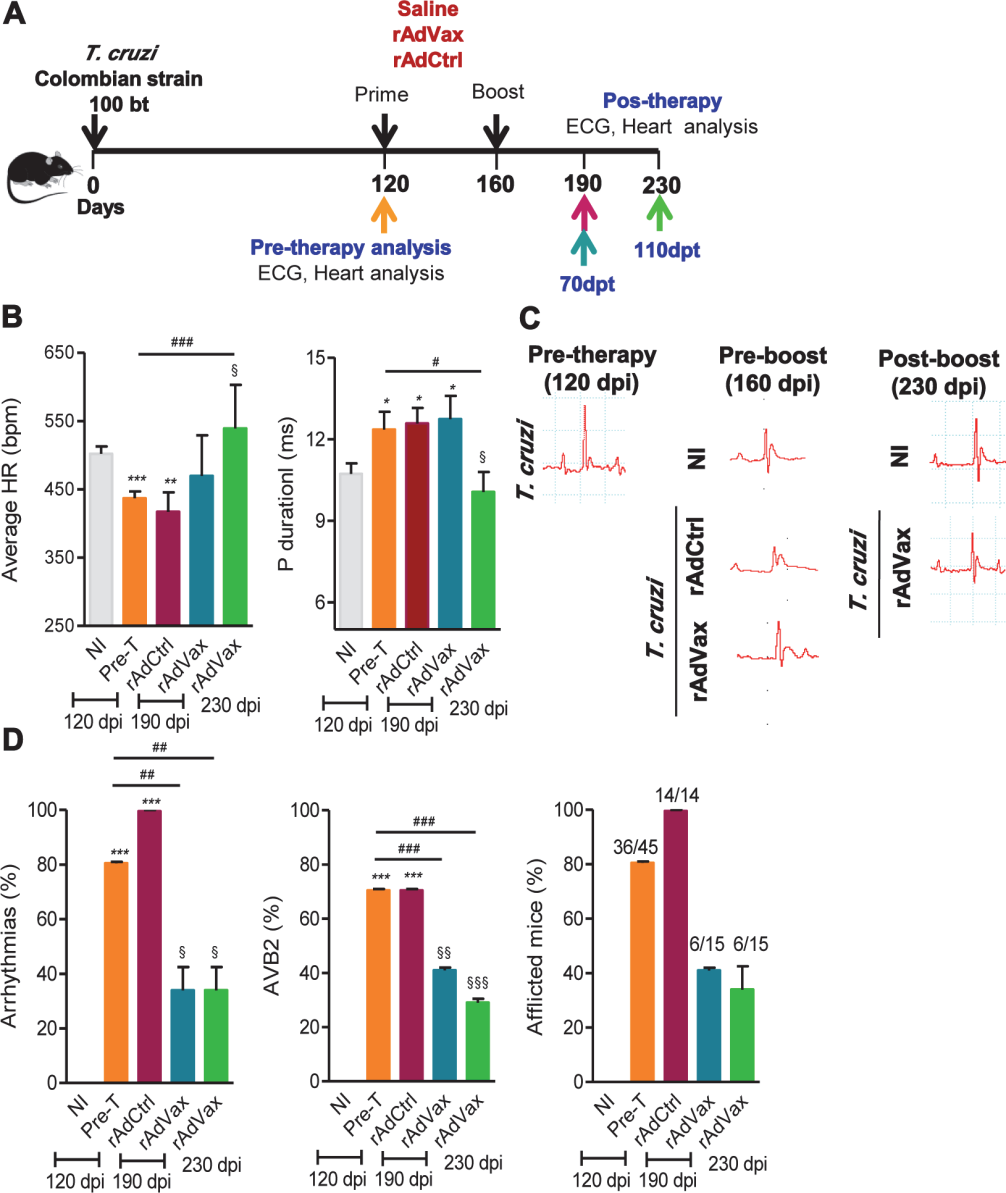
rAdVax immunotherapy ameliorated electrical abnormalities of chronically *T. cruzi*-infected mice. (**A**) Chronically Colombian-infected mice (120 dpi) were primed-boosted with 2 × 10^8^ plaque-forming units (PFU) of rAdCtrl or a mixture of 10^8^ PFU of each adenovirus vaccine preparation (rAdASP2+rAdTS; rAdVax) and analyzed for electrocardiogram (ECG) abnormalities at 70 and 110 days post-therapy (dpt). (**B**) Heart rate (beats per minutes, bpm) and P wave duration (per ms). The data are shown as the means ± SD per group of 7–8 mice. (**C**) Representative ECG register segments of sex- and age-matched noninfected (NI) controls and *T. cruzi*-infected mice injected with saline or vaccinated with rAdCtrl or rAdVax at 160 dpi (40 days post-prime) and 230 dpi (110 dpt). (**D**) Summary of the group data from NI controls and chronically *T. cruzi*-infected mice injected with saline or vaccinated with rAdCtrl or rAdVax, showing the frequency of mice presenting arrhythmias (ART), second-degree atrioventricular block (AVB2) and afflicted with ECG alterations. * *P* <0.05, **P <0.01 and ***P <0.001, experimental groups compared with NI controls. ^#^ *P* <0.05, ^##^ *P* <0.01 and ^###^ *P* <0.001, rAdVax-immunized compared with pre-therapy *T. cruzi*-infected mice. ^§^ *P* <0.05, ^§§^ *P* < 0.01 and ^§§§^ *P* <0.001, rAdVax-immunized compared with rAdCtrl-injected *T. cruzi*-infected mice.

To test the hypothesis that a vaccine might delay progression and, even, reverse CCC, chronically infected mice (at 120 dpi) were subjected to the homologous prime-boost vaccination with rAdVax (Fig. 2A). At 120 dpi, electrocardiogram (ECG) abnormalities, such as low heart rate (Fig. 2B) and prolonged P wave, were evident (Fig. 2B and 2C). Indeed, an analysis of all *T. cruzi*-infected mice showed that at 120 dpi (pre-therapy), 80% of the Colombian-infected C57BL/6 mice were afflicted with electrical abnormalities (Fig. 2D). At 150 dpi, 100% of the not-treated or saline-injected Colombian-infected C57BL/6 mice presented ECG alterations (as shown in Fig. 1E), corroborating previous data [18, 19]. At 160 dpi (40 days post-therapy initiation), 100% of rAdCtrl-injected mice presented ART and 70% presented AVB2 in a manner that 100% showed ECG abnormalities. In contrast, only 40% of rAdVax-immunized mice presented electrical abnormalities, suggesting that immunotherapy with recombinant rAdVax decreased the progression of CCC. Moreover, whereas 80% of *T. cruzi*-infected mice presented ART and other ECG abnormalities before therapy (at 120 dpi), only 40% of rAdVax-vaccinated mice presented ART and AVB2 at 230 dpi. Taken together, these data demonstrated that homologous prime-boost immunotherapy with rAdVax vaccine reversed the chronic electrical conduction abnormalities induced by infection with the Colombian strain of *T. cruzi*.

**Figure 3:**
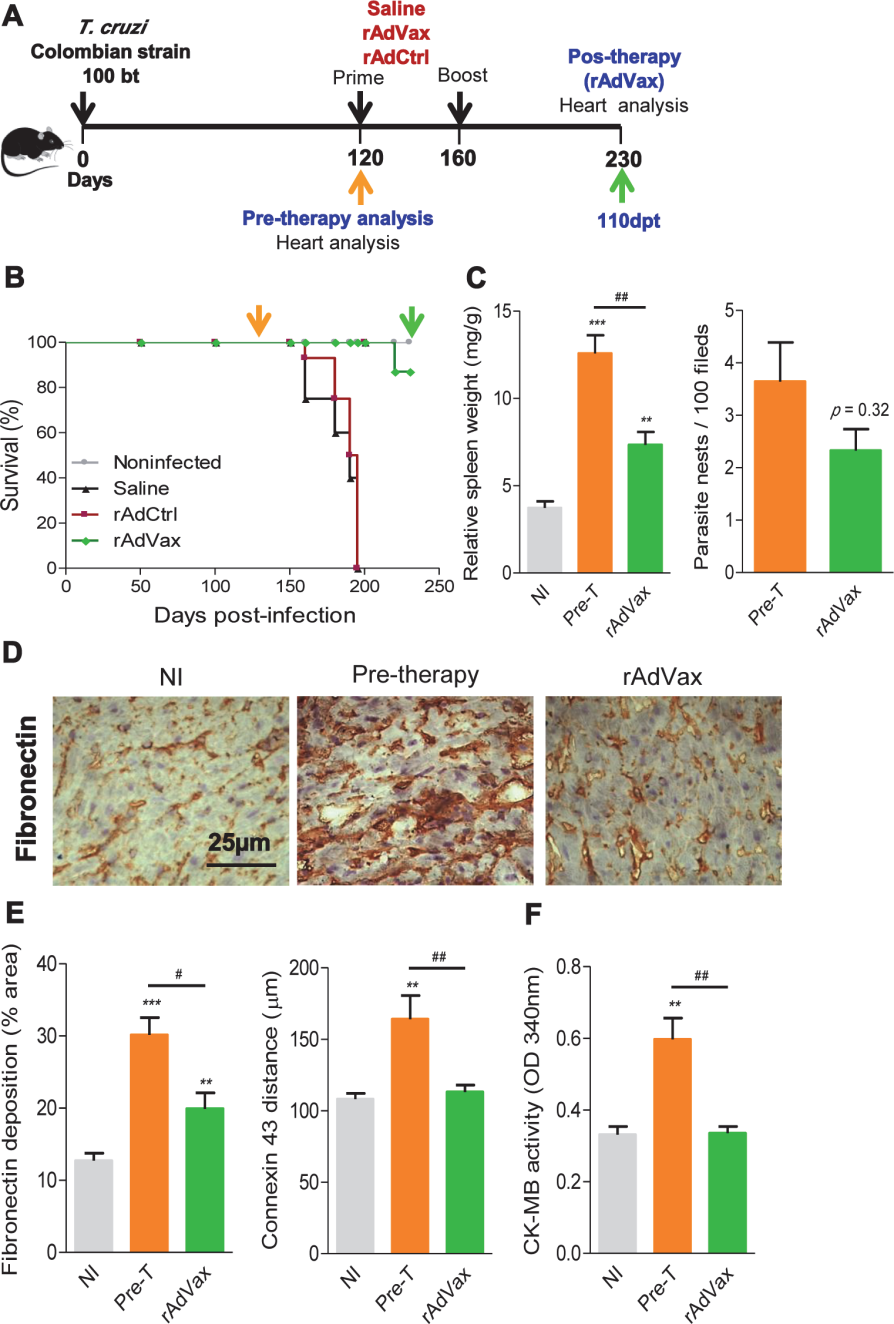
rAdVax immunotherapy recovered the injured heart tissue of chronically *T. cruzi*-infected mice. (**A**) Chronically Colombian *T. cruzi* strain-infected mice were evaluated for heart injury markers pre-therapy (120 dpi) or primed-boosted with 2 × 10^8^ plaque-forming units (PFU) of rAdCtrl or a mixture of 10^8^ PFU of each adenovirus vaccine preparation (rAdASP2+rAdTS; rAdVax). Mortality was recorded until 230 dpi (110 days post-therapy; dpt), when the surviving mice were analyzed for heart injury markers. (**B**) Kaplan-Meier curve representing the percentages of surviving mice (14–20 mice/group in two independent experiments). (**C**) Relative spleen weight (mg of spleen/g of body) and quantitative immunohistochemical staining (IHS) data for *T. cruzi* parasitism (nests/100 microscopic fields) in the heart tissue of chronically infected mice (120 and 230 dpi, respectively, pre- and post-therapy). (**D**) IHS showing fibronectin (FN)-stained areas in representative cardiac tissue sections of noninfected (NI) controls and chronically *T. cruzi*-infected mice pre- (120 dpi) and post-therapy (230 dpi; 110 dpt) with rAdVax. (**E**) Quantification of the FN-stained area (%) and connexin 43 (Cx43)-containing gap junction distances detected using IHS staining of heart tissue sections of NI controls or *T. cruzi*-infected mice pre- and post-therapy with rAdVax. (**F**) Evaluation of CK-MB activity in the serum of NI controls and *T. cruzi*-infected mice pre- and post-therapy with rAdVax. The data are presented as the means ± SD. ** *P* <0.01 and ***P <0.001, experimental groups compared with NI controls. ^#^ *P* <0.05 and ^##^ *P* <0.01, rAdVax-immunized compared with pre-therapy *T. cruzi*-infected mice.

The chronic infection of C57BL/6 mice with the Colombian strain of *T. cruzi* induces CCC, which is characterized by heart injury with connexin-43 (Cx43) disorganization and fibronectin (FN) deposition in the cardiac tissue and increased CK-MB activity in the serum [5, 18, 20]. Therefore, we tested the capacity of the homologous prime-boost rAdVax immunotherapy to reverse heart injury. To this end, C57BL/6 mice were infected and all mice were analyzed at 120 dpi (pre-therapy), when groups were formed, and the prime-boost rAdVax immunization protocol was initiated. A group of *T. cruzi*-infected mice was euthanized and the tissues were collected (pre-therapy). At 160 dpi and 40 days post-therapy, all mice were analyzed for electrical alterations, boosted and analyzed at 230 dpi, 110 days post-therapy (Fig. 3A). No difference in survival rate was observed in *T. cruzi*-infected mice that received saline or rAdCtrl and all mice in these groups were dead at 200 dpi (Fig. 3B). At 230 dpi, 87% of rAdVax-immunized mice survived (13/15), compared with 0% of rAdCtrl-inoculated (0/14) and saline-injected (0/14) mice (Fig. 3B). The surviving mice were analyzed at 230 dpi (110 days post-therapy) for heart electrical abnormalities, sacrificed and analyzed for cardiac tissue alterations compared with mice sacrificed at 120 dpi (pre-therapy). In post-therapy rAdVax-inoculated mice (at 230 dpi), there was a significant decrease in *T. cruzi*-induced splenomegaly (*P* < 0.01). Similarly to pre-therapy (120 dpi) mice, low heart parasitism persisted in rAdVax-immunized mice (Fig. 3C). In addition, pre-therapy and post-therapy parasites were rarely detected in the circulating blood (110 ± 70.2 × 10^3^ trypomastigotes/mL in pre-therapy *vs*. 22.8 ± 9.5 × 10^3^ trypomastigotes/mL in rAdVax; *P* > 0.05). Nevertheless, immunotherapy with rAdVax significantly reduced FN deposition in the cardiac tissue (Fig. 3D and 3E). Furthermore, Cx43 disorganization in the cardiac intercalary discs revealed as enhanced distance of Cx43-stained junctions, a marker of cardiomyocyte injury [21], was significantly reversed in mice treated with rAdVax compared with the pre-therapy condition (Fig. 3E). In addition, levels of CK-MB activity in the serum were lower in post-therapy mice compared with pre-therapy mice (Fig. 3F). Thus, these data support that immunotherapy with rAdVax during chronic *T. cruzi* infection ameliorated electrical abnormalities and recovered heart tissue injury.

### Recovering effects of rAdVax immunotherapy on heart tissue were associated with reduced polyclonal and cytotoxic responses but preserved parasite-specific IFNγ production

**Figure 4:**
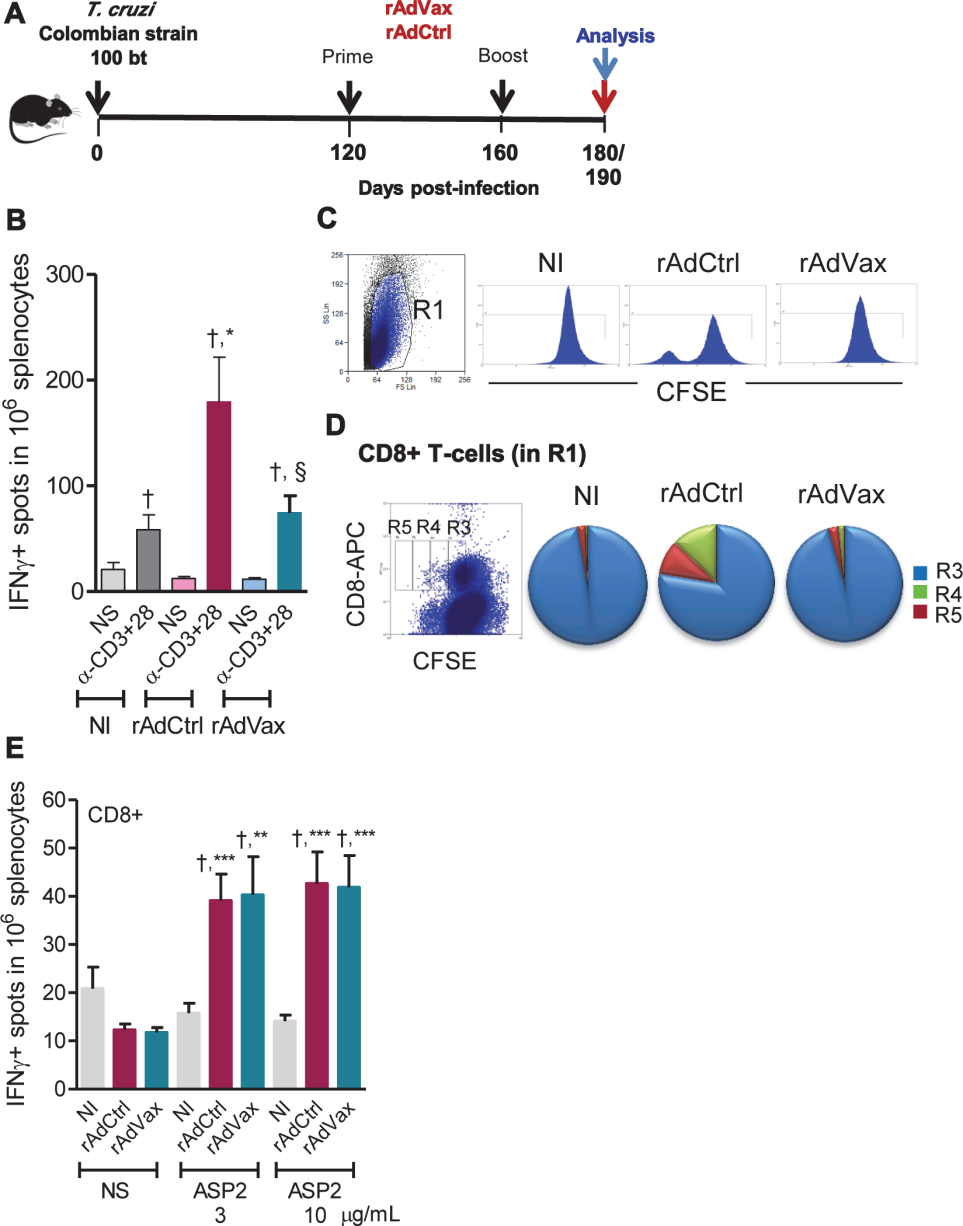
Shift of the immune response through rAdVax immunotherapy in chronically *T. cruzi*-infected mice. (**A**) Chronically Colombian-infected mice were primed-boosted with 2 × 10^8^ plaque-forming units (PFU) of rAdCtrl or a mixture of 10^8^ PFU of each adenovirus vaccine preparation (rAdASP2+rAdTS; rAdVax) and analyzed at the endpoints, as indicated. (**B**) Numbers of IFNγ-secreting cells in the spleens of immunized mice were determined using ELISpot after 20 hours of stimulation with anti-CD3 plus anti-CD28. (**C**) Representative histograms showing the R1-gated CFSE^high^-based lymphoproliferative response after 72 hours of stimulation with anti-CD3 plus anti-CD28. (**D**) Representative dot plot showing the R1-gated CFSE^Low^-based lymphoproliferative response after 72 hours of treatment with anti-CD3 plus anti-CD28. After stimulation, the spleen cells were stained with APC-conjugated anti-CD8. Cycling CFSE^Low^CD8^+^ cells were detected in gates R3, R4 and R5. (**E**) Numbers of CD8^+^IFNγ-secreting cells in the spleens of immunized mice were determined by ELISpot using the H-2K^b^-restricted VNHRFTLV peptide (3 and 10 μg/mL). The results represent three to five mice per experimental group of non-infected (NI) controls and *T. cruzi*-infected mice immunized with rAdCtrl or rAdVax and analyzed at 190 dpi (70 days post-therapy). * *P* <0.05, ** *P* <0.01 and ***P <0.001, experimental groups compared with NI controls. ^†^ *P* <0.05, non-stimulated compared with stimulated condition in an experimental group. § *P* <0.05, rAdVax-immunized compared with rAdCtrl-injected *T. cruzi*-infected mice.

Next, we investigated whether the beneficial effect of the prime-boost immunotherapy with rAdVax in chronically infected C57BL/6 mice was associated with reduction in the abnormal polyclonal activation observed in chronically infected mice and/or shift of the immune response to a protective profile. To this end, chronically infected mice received the homologous prime-boost vaccine rAdVax and were analyzed at 190 and 230 dpi, corresponding to 70 and 110 days post-therapy (Fig. 4A). As depicted in Fig. 4B, the potent IFNγ recall response after stimulation of mononuclear spleen cells with anti-CD3 plus anti-CD28, previously described as a hallmark of chronically *T. cruzi*-infected mice [22], was reproduced in the present study in the Colombian-infected rAdCtrl-immunized mice. In contrast, this response was abrogated by rAdVax immunotherapy (Fig. 4B). Moreover, the intense anti-CD3 plus anti-CD28-triggered lymphoproliferative response observed in total splenic T-cells from *T. cruzi*-infected mice injected with rAdCtrl was also inhibited by rAdVax immunotherapy (Fig. 4C). Notably, the increased anti-CD3 plus anti-CD28-triggered CD8^+^ T-cell proliferation observed during chronic infection was also reversed by rAdVax inoculation (Fig. 4D), whereas CD8^+^ T-cell recognition of the H-2K^b^-restricted VNHRFTLV ASP2 peptide was preserved in rAdVax-immunized mice (Fig. 4E).

### Beneficial effect of rAdVax therapy was associated with reduced frequency of degranulated CD107a^+^ CD8^+^ T-cells

**Figure 5:**
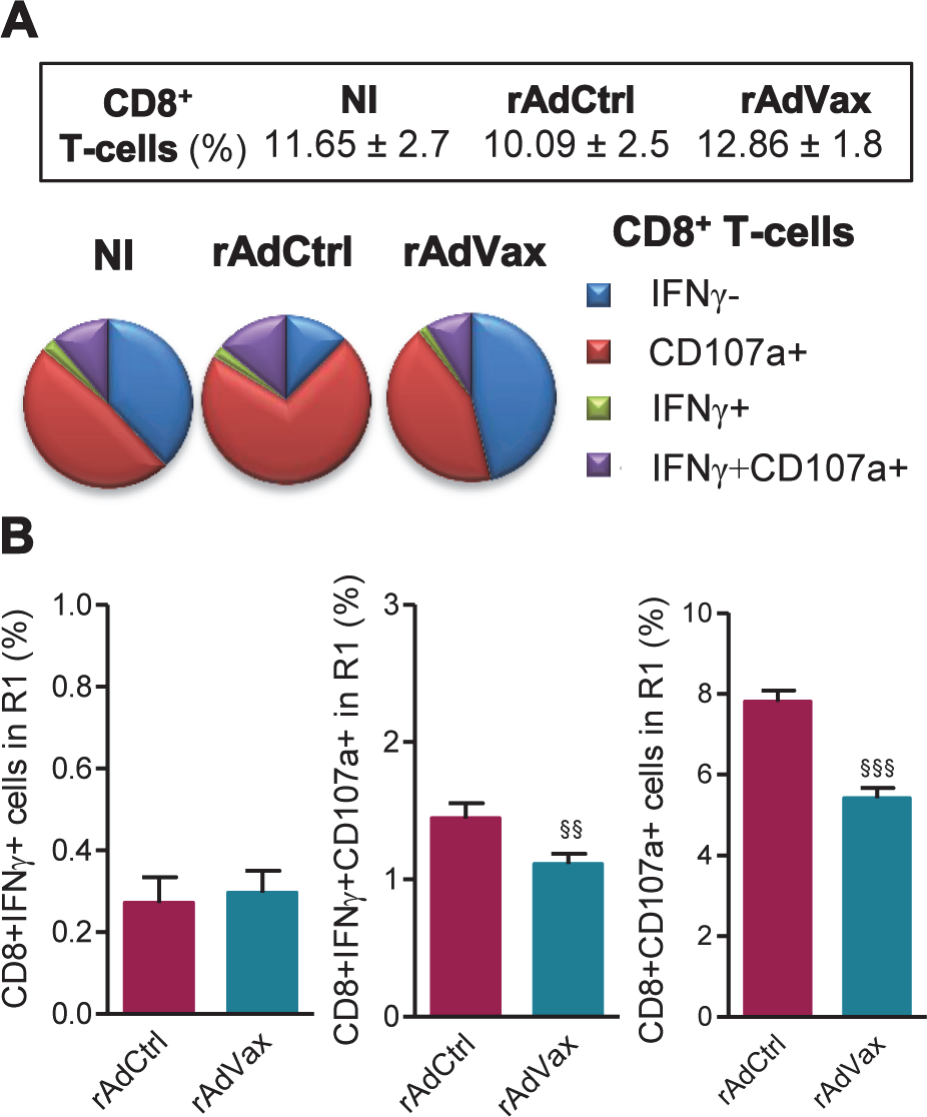
Reduced frequency of CD107a^+^CD8^+^ T-cells in chronically *T. cruzi*-infected mice subjected to rAdVax immunotherapy. Chronically Colombian-infected mice (120 dpi) were primed-boosted with 2 × 10^8^ plaque-forming units (PFU) of rAdCtrl or a mixture of 10^8^ PFU of each adenovirus vaccine preparation (rAdASP2+rAdTS; rAdVax), as experimental scheme in Fig. 2. The spleen cells were analyzed using flow cytometry at 190 dpi (70 days post-therapy). (**A**) Frequencies of total CD8^+^ T-cells (box) and mono and multifunctional IFNγ^−^CD107a^−^, CD107a^+^, IFNγ^+^ or IFNγ^+^CD107a^+^ CD8^+^ T-cells (pie charts). (**B**) Graphs showing the frequencies of IFNγ^+^, IFNγ^+^CD107a^+^ and CD107a^+^ CD8^+^ T-cells after 72 hours of *in vitro* stimulation with VNHRFTLV ASP2 peptide. The results represent three to five mice per experimental group. ^§§^ *P* < 0.01 and ^§§§^ *P* <0.001, rAdVax-immunized compared with rAdCtrl-injected *T. cruzi*-infected mice.

Then, we evaluated the frequency of CD8^+^ T-cells expressing CD107a, a marker for T-cell degranulation used to evaluate CTL activity [23]. *Ex vivo*, the frequencies of CD8^+^ T-cells in the spleen were similar in all studied groups (Fig. 5A, *box*). Compared with age-matched NI control mice, there is an increase in the proportions of CD8^+^ T-cells expressing CD107a in *T. cruzi*-infected mice injected with rAdCtrl (Fig. 5A). Importantly, this increase in the frequency of CD107a^+^ CD8^+^ T-cells was abrogated by the therapeutic rAdVax administration (Fig. 5A). Further, when *in vitro* stimulated with VNHRFTLV ASP2 peptide there is a preferential response of CD8^+^IFNγ^+^CD107a^+^ cells and CD8^+^CD107a^+^ cells in mice injected with rAdCtrl (Fig. 5B). Therapeutic rAdVax administration significantly reduced the frequency of CD8^+^IFNγ^+^CD107a^+^ cells and, moreover, diminished the frequency of CD8^+^CD107a^+^ cells recognizing the VNHRFTLV ASP2 peptide (Fig. 5B).

### The beneficial effect of the combined rAdVax therapy was associated with a protective effector profile in heart tissue

**Figure 6:**
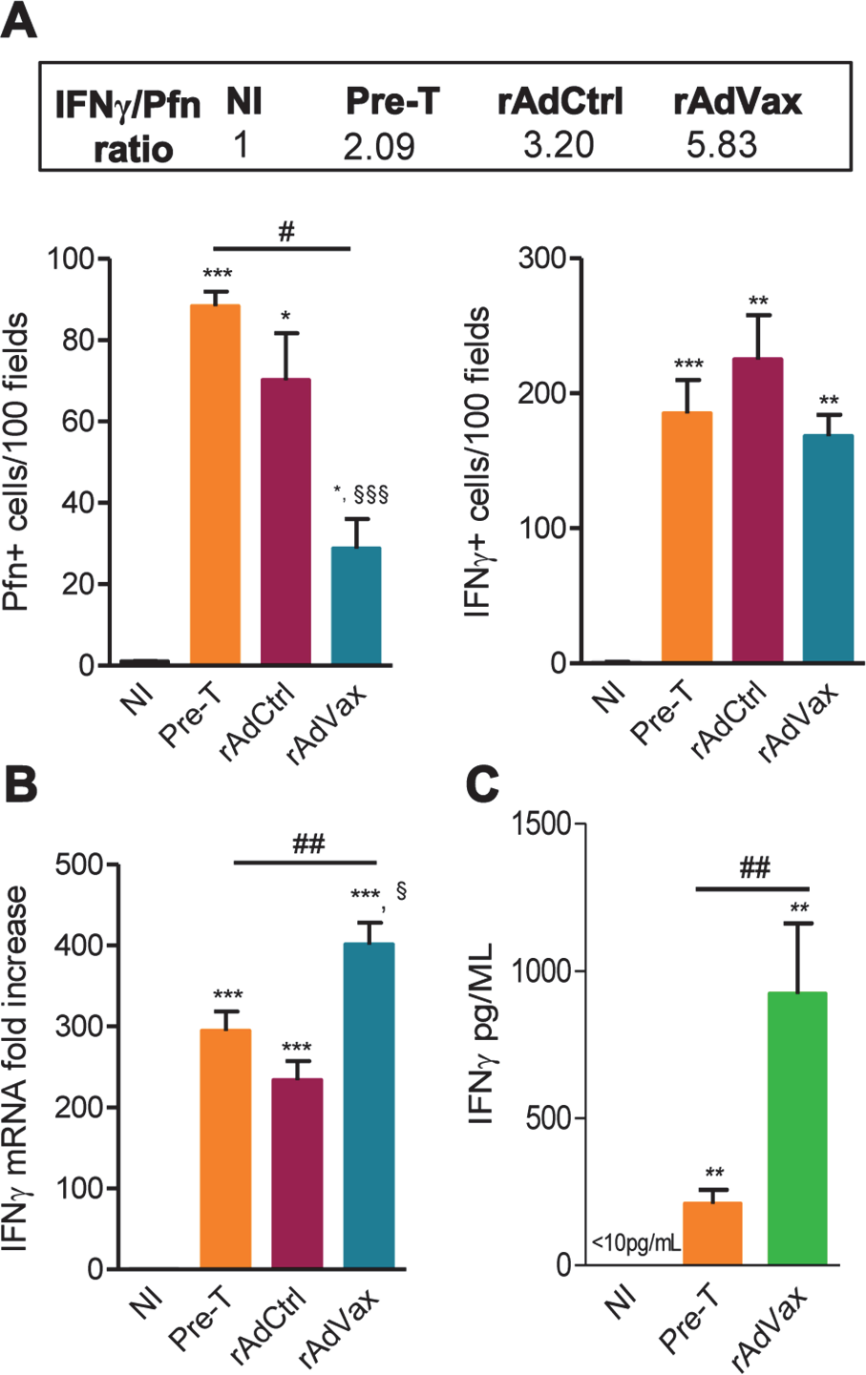
rAdVax immunotherapy increased IFNγ expression in the heart tissue and in the serum. Chronically Colombian-infected mice were immunized with 2 × 10^8^ plaque-forming units (PFU) of rAdCtrl or a mixture of 10^8^ PFU of each adenovirus vaccine preparation (rAdASP2+rAdTS; rAdVax) in a prime-boost homologous protocol. The hearts and sera of noninfected (NI) controls, infected mice pre-therapy (120 dpi) and rAdCtrl- or rAdVax-immunized mice were collected at 190 dpi (70 days post-therapy; dpt) or 230 dpi (110 dpt). (**A**) immunohistochemical staining (IHS) quantification of Pfn^+^ and IFNγ^+^ cells in the cardiac tissue; relative ratios of IFNγ^+^ /Pfn^+^ cells are shown in the upper-box. (**B**) Expression of IFNγ mRNA in the heart tissue detected by qRT-PCR. The data are expressed as the mRNA fold-increase in relation to NI controls. (**C**) Concentration of IFNγ in the serum of NI controls and *T. cruzi*-infected pre-therapy and rAdVax-immunized mice. The results represent three to five mice per experimental group. * *P* <0.05, ** *P* <0.01 and ***P <0.001, experimental groups compared with NI controls. ^#^ *P* <0.05 and ^##^ *P* <0.01, rAdVax-immunized compared with pre-therapy *T. cruzi*-infected mice. ^§^ *P* <0.05 and ^§§§^ *P* <0.001, rAdVax-immunized compared with rAdCtrl-injected *T. cruzi*-infected mice.

It was previously demonstrated that perforin^+^ and IFNγ^+^ CD8^+^ T-cells play antagonistic roles in the heart tissue of *T. cruzi*-infected mice [5]; therefore, we examined whether rAdVax immunization influenced the number of cytotoxic (Pfn^+^) and inflammatory (IFNγ^+^) cells composing the chronic *T. cruzi*-induced myocarditis. All groups of chronically *T. cruzi*-infected mice presented increased numbers of Pfn^+^ and IFNγ^+^ cells infiltrating the heart tissue compared with age-matched NI controls (Fig. 6A). However, compared with rAdCtrl-injected mice rAdVax-immunized mice showed reduced number of Pfn^+^ cells but similar number of IFNγ^+^ cells infiltrating the cardiac tissue (Fig. 6A). Moreover, therapy with rAdVax (analysis at190 dpi; 70 days post vaccine therapy) decreased the number of Pfn^+^ cells infiltrating the heart tissue compared with mice pre-therapy (120 dpi). Interestingly, the IFNγ/Pfn ratio was increased in rAdVax-immunized chronically infected mice (5.83) compared with rAdCtrl-injected mice (3.20) and pre-therapy mice (2.09) (Fig. 6A, *box*). Compared with NI control mice, a significant increase in IFNγ mRNA expression was detected in the cardiac tissue obtained from infected mice before (at 120 dpi) and after (at 190 dpi) immunotherapy with rAdCtrl and rAdVax (Fig. 6B). Remarkably, vaccination with rAdVax significantly increased the expression of IFNγ mRNA in comparison with rAdCtrl-immunized and pre-therapy mice (Fig. 6B). Therefore, these data support that rAdVax shaped the IFNγ/Pfn balance in the chronic *T. cruzi*-induced myocarditis favoring the presence of IFNγ^+^ cells and the production of IFNγ. Compared with pre-therapy chronically infected mice, rAdVax-inoculated mice exhibited no alterations in the serum concentrations of the inflammatory cytokines IL-1α, IL-2, IL-4, IL-5, IL-10, IL-17 and TNF (S4A and S4B Fig.). Interestingly, rAdVax immunization significantly increased IFNγ levels compared with pre-therapy *T. cruzi*-infected mice (S4A Fig. and Fig. 6C).

### rAdVax therapy during chronic *T. cruzi* infection was associated with reduced NO levels in the serum and iNOS in the heart tissue

**Figure 7:**
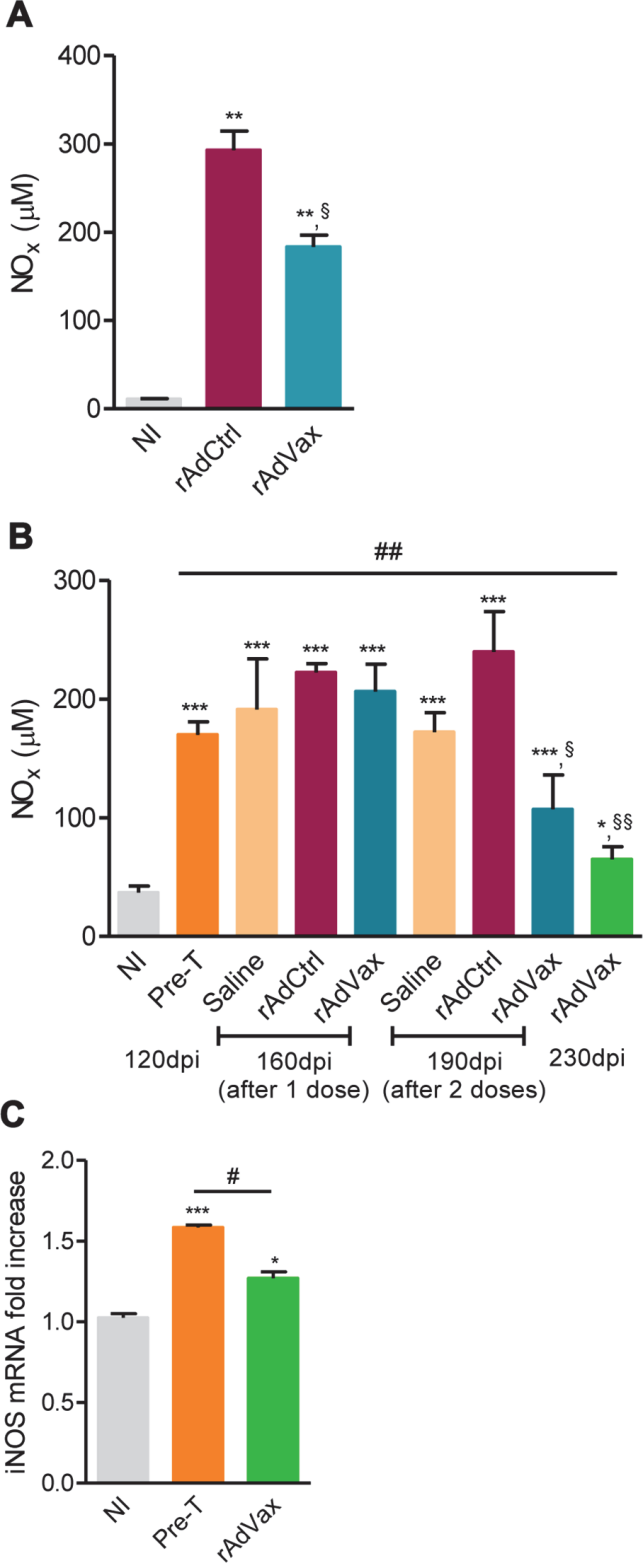
rAdVax immunotherapy modulated serum NOx levels and cardiac iNOS expression in chronically *T. cruzi*-infected mice. Chronically Colombian-infected mice were immunized with 2 × 10^8^ plaque-forming units (PFU) of rAdCtrl or a mixture of 10^8^ PFU of each adenovirus vaccine preparation (rAdASP2+rAdTS; rAdVax) in a prime-boost homologous protocol. The hearts and sera of noninfected (NI) controls, infected mice pre-therapy (120 dpi) and rAdCtrl- or rAdVax-immunized mice were collected at different time-points, accordingly with the experimental design. (**A**) Concentration of NO_x_ in the serum NI controls and infected mice at 190 dpi (70 days post-therapy; dpt). (**B**) Concentration of NO_x_ in the serum of NI controls, chronically *T. cruzi*-infected mice pre-therapy (at 120 dpi) and after immunization with rAdCtrl or rAdVax: 1 dose (at 160 dpi; 40 dpt), 2 doses (at 190 dpi; 70 dpt) and at the endpoint (post-therapy; at 230 dpi; 110 dpt). (**C**) Relative quantification of iNOS mRNA expression quantified using qRT-PCR in the heart tissue of NI controls and chronically *T. cruzi*-infected mice pre-therapy (at 120 dpi) and post-therapy with rAdVax (at 230 dpi; 110 dpt). The results represent three to seven mice per experimental group. * *P* <0.05, ** *P* <0.01 and ***P <0.001, experimental groups compared with NI controls. ^#^ *P* <0.05, rAdVax-immunized compared with pre-therapy *T. cruzi*-infected mice. ^§^ *P* < 0.05 and ^§§^ *P* <0.01, rAdVax-immunized compared with rAdCtrl-injected *T. cruzi*-infected mice.

Lastly, the NO/ iNOS pathway has been associated with heart injury and CCC severity in chronic *T. cruzi* infection [18, 24]. The kinetic study of serum NO_x_ levels in *T. cruzi*-infected mice revealed that during acute infection, NO_x_ concentrations in the serum (S5A Fig.) paralleled parasitemia (at 42–45 dpi) and higher levels of CK-MB activity in the serum (S5B Fig.). After parasite control, a reduction in NO_x_ levels was detected; however, during the chronic phase of infection, NO_x_ levels were increased and paralleled the levels of CK-MB activity in the serum (S5B Fig.). Therefore, we assessed the effect of rAdVax therapy in chronically infected mice on the concentration of serum NO_x_ and the expression of iNOS/NOS2 in cardiac tissue. All chronically *T. cruzi*-infected mice shown increased NOx levels in the serum compared with age-matched NI controls (Fig. 7A and 7B). Interestingly, in comparison with rAdCtrl-inoculated mice rAdVax-immunized mice (at 190 dpi; 30 days after the boost immunization with rAdVax) showed a significant decrease in the concentrations of NO_x_ in the serum (Fig. 7A). Furthermore, a kinetic study revealed a significant reduction in the levels of NO_x_ in the serum of rAdVax-immunized mice (190 dpi; 30 days after the boost) in comparison with rAdCtrl mice. Further, reduction of serum NO_x_ level was more prominent after two doses of rAdVax therapy, (230 dpi; 70 days after the boost immunization with rAdVax) (Fig. 7B). Moreover, there was a significant decrease in the levels of NO_x_ in the serum of mice that received two doses of rAdVax compared with pre-therapy mice (Fig. 7B). Importantly, the expression of iNOS/NOS2 mRNA was significantly reduced in the cardiac tissue of chronically infected mice immunized with two doses of rAdVax, in comparison with pre-therapy mice (Fig. 7C).

## Discussion

One of the greatest challenges in chronic Chagas’ heart disease is to develop therapies that improve prognosis and, even, reverse cardiac injury. Here we used the recombinant Type 5 adenovirus carrying sequences of the *trans*-sialidase family ASP2 and TS proteins in a homologous prophylactic or therapeutic prime-boost protocol aiming at inhibiting development and progression or reversing chronic *T. cruzi*-induced heart damage. The prophylactic rAdVax immunization successfully reduced acute heart parasitism and cardiomyocyte damage and decreased the frequency of mice afflicted by chronic electrical abnormalities due to challenge with the Colombian *T. cruzi* Type I strain. Moreover, the therapeutic administration of rAdASP2+rAdTS to chronically Colombian-infected mice afflicted by CCC decreased cardiopathy progression and, remarkably, reversed electrical abnormalities and heart tissue injury. Further investigation showed that the clinical beneficial effects were associated with reduction of the prototypical *T. cruzi*-induced polyclonal T-cell proliferation and, moreover, with reprogramed immune responses, both systemically and in the heart tissue, favoring IFNγ production and decreasing cytotoxic activity, NO_x_ production and iNOS/NOS2 expression in *T. cruzi* infection.

The prophylactic administration of rAdASP2, rAdTS or rAdASP+rAdTS combined vaccines in prime-boost protocols elicited humoral and cellular immune response [16, 17]. Vaccination of C57BL/6 (H-2^b^) with either rAdASP2 or rAdTS increased survival frequency, but only the combined rAdASP+rAdTS inoculum induced complete and long lasting protection against a challenge with the Y *T. cruzi* Type II strain parasites [16]. Further, the combined rAdASP+rAdTS vaccination of A/Sn (H-2^a^) mice was shown to be protective against a challenge with the Y and Colombia *T. cruzi* Type II strains [17]. In addition, a heterologous plasmid DNA prime-rAdHu5 boost vaccination carrying the ASP2 sequence generated a stable pool of protective long-lived effector memory CD8^+^ T-cells specific for *T. cruzi* [25]. Altogether, ASP2 and TS are shown to be suitable candidate antigens to trigger both humoral and cellular protective immunity, which are critical requisites for an immunoprophylactic protein vaccine that is presented as a pure polypeptide or within delivery vectors (plasmid, adenovirus) [13, 14, 16, 17, 25–28].

Based on the demonstration that homologous rAdVax prime-boost scheme induced antibodies and efficient IFNγ-producing and CTL CD8^+^ T-cell effectors, corroborating previous data adopting different vaccination strategies [16, 17, 27], we hypothesized that rAdVax would also induce protective response to *T. cruzi* Type I strain-induced CCC. To test this assumption, we used a low-dose (100 bt) inoculum of the Colombian strain, facilitating acute phase survival but inducing CCC [5, 18, 19]. Importantly, the prophylactic vaccination of C57BL/6 mice with rAdVax significantly reduced heart parasitism during the acute phase, which remained low during chronic infection. Moreover, prophylactic therapy with rAdVax significantly precluded heart abnormalities (including ARTs and AVBs), major clinical signs of Chagas’ heart disease [1]. Further, prophylactic therapy with rAdVax reduced the acute heart parasitism and cardiomyocyte damage induced by the CL-Brener *T. cruzi* Type VI strain. Importantly, our data support that immune responses triggered to antigen of a *T. cruzi* strain may allow resistance in a broad manner. Notably, human populations exhibit a powerful innate and acquired immune response to *T. cruzi* during acute infection, independently of the parasite strain, facilitating the development of the chronic phase of infection [1, 3, 15]. Therefore, the results of the present study corroborate our previous data showing that an association of different parasite antigens delivered in an appropriated protocol might have a beneficial impact on Chagas infection prophylaxis. Particularly considering vaccines as a strategy for decreasing parasite load in reservoirs, such as dogs, rAdVax may be a powerful tool to inhibit the domestic and peri-domestic cycle of *T. cruzi*, regardless the parasite strain faced in the natural infection [6]. Moreover, the prophylactic administration of rAdVax precluded relevant aspects of chronic Chagas’ heart disease [1], therefore surging as a strategy to improve prognosis.

Millions of people are infected with *T. cruzi*. Among these, 20–40% will manifest the cardiac form of CD, with mild to severe signs and symptoms and premature death, within 10–30 years after infection [1, 7]. Therefore, therapeutic vaccines may emerge as a tool to improve prognosis of chronic patients. The present study is the first to show that a vaccine preparation may abrogate, delay progression and, moreover, reverse *T. cruzi*-induced ECG alterations, including bradycardia, ARTs and AVB2. These electrical abnormalities represent important features detected in cardiophatic chagasic patients [1] recapitulated in the model of chronically *T. cruzi*-infected mice used [5, 18,19]. Increased levels of CK-MB activity in serum, a biomarker of cardiomyocyte lesions [29], that is consistently detected in chronically infected mice [5, 20], was also reversed by rAdVax immunization of chronically infected mice. Furthermore, two main heart tissue injuries of chronically Colombian-infected mice, i.e. enhanced FN deposition and Cx43 disorganization and loss [18, 20], were reversed through therapeutic immunization with rAdVax. Chronic cardiac fibrosis, revealed as deposition of extracellular matrix components including FN, is associated with CD severity in patients [30, 31], non-human primates [24] and mice [18]. Here we describe that chronically infected mice show overdeposition of FN in the cardiac tissue, which is reversed by therapeutic administration of rAdVax. In benznidazole-treated mice, reduced heart parasitism is accompanied by decreased FN deposition and fibrosis [32]. Furthermore, therapeutic intervention in the inflammatory axis with the CCR1/CCR5 antagonist Met-RANTES [20] and the anti-tumor necrosis factor Infliximab antibody also reduced FN deposition in the cardiac tissue of chronically Colombian-infected mice [19], supporting that this a reversible feature of CCC. The disorganization and loss of Cx43, the most abundant ventricular gap junction protein, is associated with arrhythmogenic disease [21]. This is another important biomarker of heart tissue damage associated with severity of electrical abnormalities in *T. cruzi*-infected mice [18]. Moreover, cardiac Cx43 expression is down-regulated in patients with CCC [33] and in cardiopathic compared with indeterminate chronically infected rhesus monkeys [24]. The intensity of C43 loss was also associated with disease severity in models of severe and mild CCC [18]. Therefore, a major beneficial effect of the immunotherapy with rAdVax was the restoration of Cx43 expression in the heart of chronically infected mice. Taken together, these data support the idea that *T. cruzi*-induced chronic heart damage can be ameliorated after appropriated interference, which may consist of targeting the parasite [32] or other putative pathogenic factors, such as chemotactic [20] or inflammatory [19] mediators. Hence, these findings reinforce the idea that vaccination with rAdVax (rAdTS+rAdASP2) is a promising tool for intervention in patients affected by CCC.

Taken together, these data encouraged further studies on the mechanisms by which rAdVax administration exerts beneficial effects in chronically *T. cruzi*-infected mice. Prophylactic rAdVax administration efficiently reduced acute heart parasitism, corroborating previous attempts using prophylactic vaccines carrying different immunogenic constructions, formulations and vectors that induce effective vaccines that reduce parasitism [8, 9, 16, 17, 27]. Further, prophylactic rAdVax also reduced the number of CD8^+^ cells infiltrating the heart tissue. There is a relation between parasite load and CD8^+^ cell infiltrating the heart tissue [34]. Therapeutic rAdVax immunization of chronically infected mice had no significant effect on the already low parasite load in the heart tissue. There is no relation between parasite load and myocarditis intensity in the chronic phase of infection [35], when parasites are mostly intracellular and, therefore, less accessible to effector immune response. Recently, we showed that both IFNγ^+^ and Pfn^+^ inflammatory cells present in the cardiac tissue contribute to parasite control; however, Pfn^+^ cells also contribute to tissue injury [5]. Importantly, the number of Pfn^+^ cells was reduced after rAdVax immunization of chronically infected mice, associated with improvement of heart ECG abnormalities and cardiac damage. Undoubtedly, one cannot rule out the possibility that factors of the immune response, as cytokines not modulated by rAdVax immunization as TNF, shown to promote parasite growth [36], also contribute to maintain low parasite load in vaccinated mice. Nevertheless, more than parasite load and intensity of myocarditis, the total scenario, which also includes effector cells and cytokine milieu contributing to immunological unbalance, may play crucial role in chronic Chagas’ heart disease pathogenesis [19, 20, 37].

Splenomegaly, associated with increased cellularity, is a hallmark of chronic experimental CD [5, 22]. Reversion of splenomegaly in rAdVax-treated chronically *T. cruzi*-infected mice was the first indication that this therapeutic tool interferes with parasite-triggered immunological abnormalities. Chronically *T. cruzi*-infected mice show an intense proliferative response of CD8^+^ T-cells and increased frequency of IFNγ-producing CD8^+^ T-cells after polyclonal stimulation with anti-CD3 plus anti-CD28 [22]. Here we reproduced this abnormality in rAdCtrl-injected chronically Colombian-infected mice. Importantly, CD8^+^ T-cell proliferation and IFNγ production induced by polyclonal activation with anti-CD3 plus anti-CD28 were significantly reduced in rAdVax-immunized mice. However, the *T. cruzi*-specific IFNγ-producing CD8^+^ T-cells present in chronically *T. cruzi*-infected mice were preserved in these mice. Therefore, the therapy of chronically infected mice with rAdVax in a prime-boost homologous protocol reduced the *T. cruzi*-induced chronic polyclonal activation of CD8^+^ T-cells, but preserved the potentially beneficial IFNγ-producing CD8^+^ T-cells [5].

Previous studies with vaccines have provided evidence that prophylactic vaccines using ASP2 and/or TS constructs in different delivery tools stimulate both IFNγ producers and cytotoxic CD8^+^ T-cells, which play a protective role after challenge with virulent *T. cruzi* strains [16,17,27,28,38,39]. Our results demonstrated that therapeutic homologous prime-boost vaccination with rAdVax significantly reduced the frequency of *ex vivo* freshly isolated and VNHRFTLV ASP2 peptide-specific CD8^+^CD107a^+^ T-cells. CD107a marks degranulation, paralleling the cytotoxic activity of T-cells [23]. Recent data have suggested that the induction of multifunctional CD8^+^CD107a^+^ T-cells co-expressing IFNγ might be beneficial against acute *T. cruzi* infection [40]. The data obtained in the present study, however, suggest that the persistence of CD107a^+^ T-cells during chronic infection might be detrimental. Indeed, the down-modulation of the frequency of CD8^+^CD107a^+^ T-cells paralleled the improvement of heart ECG abnormalities and tissue injury in rAdVax-immunized mice, thereby reinforcing the idea that cytotoxic CD8^+^ T-cells are detrimental in chronic *T. cruzi* infection [5]. These findings led us to inquire whether this effect of rAdVax therapy impacted the immunological status of the cardiac tissue. Indeed, rAdVax administration to chronically infected mice reduced the number of Pfn^+^ cells colonizing the cardiac tissue, whereas the numbers of IFNγ^+^ cells persisted elevated. Truly, the IFNγ^+^/Pfn^+^ cells ratio was significantly increased in rAdVax-inoculated mice. Furthermore, the expression of IFNγ mRNA in the cardiac tissue was significantly increased in rAdVax-immunized mice in comparison with rAdCtrl-injected and pre-therapy mice. Also, a remarkable increase in IFNγ levels in serum was detected in rAdVax-immunized chronically infected mice. Importantly, systemically and in heart tissue increased IFNγ expression paralleled the amelioration of ECG abnormalities and cardiac tissue damage. Again, these results are consistent with the idea that IFNγ plays a beneficial role in the heart tissue injury during chagasic infection [5]. This, immunization with vaccine constructs carrying the coding sequences of ASP2 and TS parasite antigens in an appropriate delivery system shifted the immune response to a less detrimental (cytotoxic) profile and/or to a more beneficial (IFNγ) status, which may contribute to improve the experimental Chagas’ heart disease. Altogether, these data support the idea that parasite-triggered immunological unbalance promotes Chagas’ heart disease [5, 19,41, 42].

Chronic chagasic cardiomyopathy is undoubtedly the most severe form of CD [1]. The pathogenic factors leading to CCC remain largely unknown; therefore, the comprehension of these factors and the identification of biomarkers of prognosis and severity might contribute to the development of vaccines and more efficient therapies. In this context, studies have shown that NO levels in serum parallels CCC severity in humans [43] and non-human primates [24]. Indeed, here we demonstrated that in chronic experimental CD the levels of NO_x_ paralleled CK-MB activity in serum, reinforcing the idea that NOx levels is associated with cardiomyocyte lesion [18,24]. In *T. cruzi* infection, NO is produced via iNOS/NOS2 in a wide range of cells and tissues and acts as an important trypanocidal agent [44]; however, NO can be cytotoxic and destructive to tissues when produced at high concentrations and for long periods [43]. Chagas’ heart disease relies on a complex host-parasite interrelationship. In this sense, it has been shown that *T. cruzi* persistence can serve as a stimulus for continuous iNOS/NOS2 expression in cardiac tissue and, consequently, a large amount of NO might accumulate in this tissue [45]. Hence, an increased expression of iNOS/NOS2 in heart tissue and enhanced supply of NO could lead to cardiomyocyte lesion and heart injury [24]. In the present study, chronic *T. cruzi* infection (at 120 dpi) was accompanied by a significant increase in serum NO_x_ levels and the enhanced expression of iNOS/NOS2 in heart tissue, both paralleling the heart tissue injury (Cx43 loss and FN overdeposition) and electrical alterations. Consistently, NO_x_ levels in serum and iNOS/NOS2 expression in cardiac tissue were successfully reduced after prime-boost therapy with rAdVax, paralleling amelioration of ECG abnormalities and heart tissue damage. Thus, delivery of ASP2 and TS genes in the form of rAd constructs emerges as a rational alternative tool to reprogram immune responses to a less detrimental and a more protective profile (particularly in heart tissue), interrupt progression and recover tissue injury in chronic Chagas’ heart disease.

Here we brought insights on the biological processes contributing to beneficial effects of the prime/boost with rAdVax on clinical signs of chronic experimental CD. However, the molecular mechanisms for the immunological reprograming mediating the effects of our vaccine constructions and regimen of administration were not addressed in the present study. Our data support that rAdVAx reverses several immunological abnormalities (polyclonal T-cell activation, increased frequency of CD107a^+^CD8^+^ T-cells, accumulation of Pfn^+^ cells in the heart tissue and high NO levels in the serum), thus it is conceivable that distinct molecular mechanism may take part in the reprogramming of these biological alterations, opening a new avenue to be explored.

Lastly, CD is a major public health problem in Latin America; therefore, a vaccine would be an important tool to improve the control of CD [46] and, mainly, to interfere with the outcome of CD. Here we propose the use of a recombinant AdHu5-based vaccine carrying ASP2 and TS *T. cruzi* sequences to decrease acute parasite load and prevent CCC and as an alternative to reprogram the immunological unbalance in chronically infected individuals to delay progression and, potentially, reverse prototypical pathological aspects of CCC. We consider that the main theoretical barriers (safety and immunogenicity) to use recombinant AdHu5-based ASP2/TS vaccine in chronic chagasic patients were recently overcame. Indeed, vaccine constructs for HIV and tuberculosis using recombinant AdHu5 as vector enlightened our knowledgement revealing that the vaccine regimen was safe and had an acceptable side-effect profile [47]. Moreover, the widely perceived negative effect of preexisting anti-AdHu5 immunity may not be universally applied to all AdHu5-based vaccines. Preexisting neutralizing antibodies did not affect the immunogenicity of an AdHu5-based malaria vaccine in humans [48]. Further, an AdHu5-vectored vaccine engineered to express the immune dominant *M. tuberculosis* antigen Ag85A was shown to be safe and robustly immunogenic, supporting a lack of correlation between preexisting anti-AdHu5 neutralizing antibody titers and the magnitude of vaccine-induced T-cell activation [49]. Therefore, the AdHu5-based ASP2/TS therapeutic vaccine administered either alone or combined with trypanocidal drugs as benznidazole [1] or target-based adjuvants as rapamycin [50], might improve the prognosis of CCC, providing a new approach for the development of useful protocols to treat CD patients. Therefore, the results obtained here demonstrate that the development of a successful vaccine for CD is not beyond reach [2,7]. However, one cannot ignore that with success will come the economic barriers to produce and deliver a vaccine against a neglected disease. Further, the extemporaneous campaigns nurturing refractivity to vaccines may emerge as a new challenge to be surpassed.

## Materials and Methods

### Ethics statement

This study was carried out in strict accordance with the recommendations in the Guide for the Care and Use of Laboratory Animals of the Brazilian National Council of Animal Experimentation (http://www.cobea.org.br/) and the Federal Law 11.794 (October 8, 2008). The Institutional Committee for Animal Ethics of Fiocruz (CEUA-Fiocruz-L004/09) and the Brazilian Biosafety National Committee (CQB/CTNBio-105/99) approved all experimental procedures used in the present study. The Experimental Animal Facility is fully accredited by the National Technical Commission on Biosafety (CTNBio; last notification, October 27, 2010). All presented data were obtained from four (INCTV1-4) independent experiments (Experiment Register Books #3 and #4, LBI/IOC-Fiocruz).

### Experimental infection

Mice obtained from the animal facilities of the Oswaldo Cruz Foundation (CECAL/Fiocruz, Rio de Janeiro, Brazil) were housed under specific pathogen-free conditions in a 12-h light-dark cycle with access to food and water *ad libitum*. Five- to seven-week-old female C57BL/6 (H-2^b^) mice were intraperitoneally (i.p.) infected with 100 blood trypomastigotes (bt) of the Colombian *T. cruzi* Type I strain or with 1000 bt of the CL-Brener *T. cruzi* Type VI strain [15] that had been prepared by passage through C57BL/6 every 35 or 10 days, respectively. Parasitemia was used as a parameter to establish acute and chronic phases [5] and mortality was recorded weekly. Sex- and age-matched noninfected (NI) controls were analyzed in parallel. Accordingly to experimental designs, groups of mice were sacrificed under anesthesia (100mg/Kg ketamine associated with 5mg/Kg xylazine chloride).

### Recombinant adenovirus construction and immunization

The construction of the replication-deficient human Type 5 recombinant adenoviruses carrying the *Escherichia coli* β-galactosidase (rAdCtrl), the mouse Ig k chain SP fused to the ASP2 1–694 amino acid coding sequence (rAdASP2) and the signal peptide and catalytic domain amino acid 34–678 of TS (rAdTS) coding sequences of the Y *T. cruzi* strain have been previously reported [16]. Mice were inoculated subcutaneously (s.c.) in the tail base with 100 μL of viral suspension comprising apyrogenic saline (BioManguinhos, Brazil) supplemented with 1% normal mouse serum (Sigma, USA) containing 2 × 10^8^ plaque-forming units (PFU) of rAdLacZ or a mixture of 10^8^ PFU of each adenovirus vaccine preparation (rAdASP2+rAdTS). The mice were immunized twice at four- to six-week intervals, as shown in the scheme of the figures. As experimental groups were analyzed at different moments, we associated the colors of the arrows indicating the moments of analyses in the experimental schemes with the colors of the bars representing the results of the analyzed groups in the figures. Additionally to experiments to analyze the immune response, in two experiments mice were immunized as described above and death was weekly registered. The results of these experiments were combined to establish survival curve.

### Reagents and antibodies

For T-cell functional assays, we used the H-2K^b^-restricted VNHRFTLV peptide from ASP2 [13] synthesized by GenScript USA Inc. (USA). For in vivo cytotoxicity assays, target and control cells were tagged with the fluorogenic dye carboxyfluorescein diacetate succinimidyl ester (CFSE, Molecular Probes, USA). For lymphoproliferation assays, we used a CFSE-based cell tracer for flow cytometry (CellTrace Cell Proliferation kit, Invitrogen, USA). For immunohistochemical staining (IHS), the polyclonal antibody against *T. cruzi* antigens and supernatants containing anti-mouse CD8a (clone 53–6.7) and anti-mouse CD4 (clone GK1.5) were produced in our laboratory (LBI/IOC-Fiocruz, Brazil). Other antibodies included an anti-F4/80 polyclonal antibody (Caltag, USA), polyclonal rabbit anti-connexin 43 (Cx43) (Sigma, USA), polyclonal rabbit anti-mouse fibronectin (FN) (Gibco-BRL, USA), biotinylated anti-rat immunoglobulin (Dako, Denmark) and biotinylated anti-rabbit immunoglobulin and peroxidase-streptavidin complex (Amersham, UK). The monoclonal antibodies anti-mouse Pfn (CB5.4, Alexis Biochemicals, USA) and anti-IFNγ (R4-6A2, BD PharMingen, USA) produced in rat were also used in IHS. For flow cytometry, PECy7-anti-CD3 (clone 17A2), APC-conjugated anti-mouse CD8a (clone 53–6.7), PerCP-anti-CD4 (clone GK1.5) and PECy-7-conjugated anti-IFNγ (clone XMG1.2) were purchased from BD Pharmingen (USA). PE-conjugated anti-CD107a (clone eBIO1D4B) was obtained from eBioscience. Endotoxin-free purified anti-CD3 (clone 145-2C11) and anti-CD28 (clone 37.51) were purchased from Southern Biotech (USA). Appropriate controls were prepared by replacing the primary antibodies with the corresponding serum, purified immunoglobulin or antibody isotype. All antibodies and reagents were used according to the manufacturers’ instructions.

### Antibody detection using ELISA

Recombinant TS or ASP2 proteins were produced in *E. coli* and total antibodies (IgM+IgG) against these proteins were detected using an enzyme-linked immunosorbent assay (ELISA) as previously described [51]. Each serum sample was serially diluted (1:200, 1:400 and 1:800) for analysis. The optical density (OD) at 405 nm higher than twice the OD detected in serum of non-immunized mice (in this case higher than 0.1) was considered positive for antibody detection. The results are presented as the mean OD of six to eight mice per group.

### IFNγ enzyme-linked immunospot (ELISpot) assay

The ELISpot assay for the enumeration of IFNγ-producing cells was performed in triplicate as previously described [39]. Plates were coated with anti-mouse IFNγ (clone R4-6A2; BD PharMingen, USA) antibody diluted in PBS (5 μg/mL). Antigen-presenting cells were primed with total *T. cruzi* antigens (10 μg/mL) for 30 minutes at 37°C. Concanavalin A (ConA, 5 μg/mL) was used as a mitogenic stimulant. After incubation, the freshly isolated splenocytes were seeded at 5 × 10^5^ cells/well and incubated with the ASP2 H-2K^b^-restricted VNHRFTLV peptide [13] for 20 hours at 37°C and 5% CO_2_. Biotin-conjugated anti-mouse IFNγ antibody (clone XMG1.2; BD PharMingen, USA) was used to detect the captured cytokines. Spots were revealed after incubation of the samples with a solution of alkaline phosphatase-labeled streptavidin (BD PharMingen, USA) and a solution of NBT (Sigma, USA) and BCIP (Sigma, USA) in Tris buffer (0.9% NaCl, 1% MgCl_2_, 1.2% Tris in H_2_O). The mean number of spots in triplicate wells was determined for each experimental condition and the number of specific IFNγ-secreting T-cells was calculated by estimating the stimulated spot count/10^6^ cells using a CTL OHImmunoSpot A3 Analyzer (USA).

### CFSE-based lymphoproliferative response

The lymphoproliferative response was assessed as described previously [5]. Briefly, spleens were removed from NI or *T. cruzi*-infected vaccinated mice and single-cell suspensions of splenocytes were prepared. The red blood cells were lysed using ACK buffer (Sigma, USA) and mononuclear cells were labeled with CFSE at a final concentration of 7 μM (CFSE^high^) or 0.5 μM (CFSE^low^). The cells were incubated in RPMI medium supplemented with 10% SBF in the presence of anti-CD3 and anti-CD28 (3 μg/mL) or 2.5 μM of the VNHRFTLV ASP2 peptide for 72 hours at 37°C and 5% CO_2_. CFSE^high^ cells were washed and fixed with 1.0% paraformaldehyde. CFSE^low^ cells were washed and labeled with APC-conjugated anti-CD8 antibody as described above and fixed using 1.0% paraformaldehyde. All samples were acquired using a Beckman Coulter CyAn 7 Color flow cytometer (Fullerton, CA, USA) and analyzed using the Summit v.4.3 Build 2445 program (Dako, Denmark).

### In vivo cytotoxicity assay

For the in vivo cytotoxicity assays, spleens collected from naïve C57BL/6 mice were treated with ACK buffer (Sigma, USA) to lyse the red blood cells. The cells were divided into two populations and labeled with the fluorogenic dye CFSE (Molecular Probes, USA) at a final concentration of 10 μM (CFSE^high^) or 0.1 μM (CFSE^low^). CFSE^high^ cells were coated with 2.5 μM of the VNHRFTLV ASP2 peptide [13] for 40 minutes at 37°C. The CFSE^low^ cells remained uncoated. Subsequently, the CFSE^high^ cells were washed and mixed with equal numbers of CFSE^low^ cells before intravenous injection (1–2 × 10^7^ cells per mouse) into C57BL/6 recipients sedated with diazepam (20 mg/Kg). Spleen cells were collected from the recipient mice at 20 hours after adoptive cell transfer as indicated in the figure legends and fixed using 1.0% paraformaldehyde. All samples were acquired using a Beckman Coulter CyAn 7 Color flow cytometer (Fullerton, CA, USA) and analyzed using the Summit v.4.3 Build 2445 program (Dako, Denmark). The percentage of specific lysis was determined using the following formula:

$$[1-\frac{\left(\%\;{\mathrm{CFSE}}^{\mathrm{high}}\mathrm{infected}/\;\%\;{\mathrm{CFSE}}^{\mathrm{low}}\mathrm{infected}\right)}{\left(\%{\mathrm{CFSE}}^{\mathrm{high}}\mathrm{NI}/\%\;{\mathrm{CFSE}}^{\mathrm{low}}\mathrm{NI}\right)}]\times 100\%$$

### Immunohistochemistry

The mice were euthanized under anesthesia and their hearts were removed, embedded in tissue-freezing medium (Tissue-Tek, Miles Laboratories, USA) and stored in liquid nitrogen. The phenotypes of the inflammatory cells (CD4^+^, CD8^+^, F4/80^+^ ), IFNγ^+^ and Pfn^+^ cells colonizing the heart tissue and the *T. cruzi* parasitism were characterized and analyzed as previously described [5]. The FN- and Cx43-positive areas in 25 fields (12.5 mm^2^) per section (three sections per heart) were evaluated using a digital morphometric apparatus as previously described [20]. The resulting images were digitized using a color view XS digital video camera adapted to a Zeiss microscope and analyzed using AnalySIS AUTO software (Soft Imaging System, USA). The data are presented as the percent positive area in the heart, the distance (μm) between stained gap junctions or the numbers of parasite nests or cells per 100 microscopic fields (400×).

### Detection of cardiac muscle creatine-kinase isoform

The activity of the creatine kinase cardiac MB isoenzyme (CK-MB) was measured as a cardiomyocyte lesion marker [29] using a commercial CK-MB Liquiform kit (Labtest, Brazil) according to the manufacturer’s recommendations, as previously adapted for mouse samples [5].

### Flow cytometry analysis

The spleens were minced and the red blood cells were removed using lysis buffer (Sigma, USA). The splenocytes were labeled and events were acquired using a CyAn-ADP Analyzer (Beckman Coulter, USA). The data were analyzed using the Summit v.4.3 Build 2445 program (Dako, USA) as described elsewhere [5].

### Phenotype of VNHRFTLV ASP2 peptide-stimulated CD8^+^ T-cells

The VNHRFTLV ASP2 peptide-specific response was assessed as described previously [5]. Briefly, spleens were removed and processed as described above. The cells were incubated in RPMI medium supplemented with 10% SBF in the presence of 2.5 μM of the VNHRFTLV ASP2 peptide for 72 hours at 37°C and 5% CO_2_. Cells were washed, processed and analyzed for flow cytometry as described above.

### Electrocardiogram (ECG) registers

The mice were sedated with diazepam (10 mg/kg) and transducers were placed subcutaneously (DII). The traces were recorded for 2 minutes using the digital Power Lab 2/20 System connected to a bio-amplifier at 2 mV for 1 second (PanLab Instruments, Spain). The filters were standardized to 0.1–100 Hz and the traces were analyzed using Scope software for Windows V3.6.10 (PanLab Instruments, Spain). ECG parameters were analyzed as previously described [5].

### Cytokine quantification

Cytokines (IL-1α, IL-2, IL-4, IL-5, IL-6, IL-10, IL-17, GM-CSF, TNF, IFNγ) were detected in serum using the commercial Th1/Th2 10plex FlowCytomix kit (MNS820FF; Bender MedSystems Inc., Austria) according to the manufacturer’s instructions. The samples were assayed with suitable controls provided by manufacturer for the construction of standard curves. The fluorescence produced by the beads was measured on a FACSCalibur flow cytometer (BD, Biosciences, USA) and analyzed using the software contained in the kit.

### NO quantification

Nitrate and nitrite (NO_x_) were determined to estimate the nitric oxide (NO) levels in the serum samples using Griess reagent and vanadium chloride III; a standard curve of 0.8 to 100 mM NaNO_2_ and NaNO_3_ was prepared as described elsewhere [24].

### Real-time quantitative RT-PCR for IFNγ and iNOS mRNA

For real-time quantitative RT-PCR (RT-qPCR), the mice hearts were harvested, washed to remove blood clots, weighed and frozen in RNAlater (#AM7021, Life Technologies, USA). Total RNA (for gene expression studies) was extracted using TRI-Reagent (Sigma, USA). All reverse transcriptase reactions were performed using a SuperScript III Kit (# 18080-051) and RT-qPCR was performed using TaqMan gene expression assays for IFNγ (#Mm01168134_m1), inducible NO synthase (iNOS/NOS2; #Mm00440502_m1) and the endogenous housekeeping control genes glyceraldehyde 3-phosphate dehydrogenase (GAPDH; #Mm99999915-g1) and β actin (#Mm00607939-s1), which were purchased from Life Technologies (USA). The reactions were performed and analyzed as previously described [52].

### Statistical analysis

The data are expressed as arithmetic means ± standard deviation. Student *t* tests, ANOVA and other appropriate tests were used to analyze the statistical significance of the observed differences. The Kaplan-Meier method was used to compare the survival times of the studied groups. All statistical tests were performed using GraphPad Prism. Differences were considered statistically significant when *P* <0.05.

## Supporting Information

S1 Fig Immunization of C57BL/6 mice with rAdVax induced specific antibodies and T-cell response. (**A**) Mice were s.c. primed-boosted with 2 × 10^8^ plaque-forming units (PFU) of rAdCtrl or a mixture of 10^8^ PFU of each adenovirus vaccine preparation (rAdASP2+rAdTS; rAdVax) or the vehicle control saline at 6-week intervals. (**B**) Total (IgM+IgG) antibodies to ASP2 and TS peptides were detected using ELISA. Each serum sample was serially diluted (1:200, 1:400 and 1:800) before analysis. The data are presented as the means of three to five mice serum samples per group. (**C**) Representative histogram of in vivo cytotoxicity assays of normal splenocytes labeled with two concentrations of CFSE (CFSE^High^ and CFSE^Low^) that were inoculated intravenously into the tail vein of immunized mice. CFSE^High^ cells were pulsed with the ASP2 peptide. CFSE^Low^ cells were unpulsed and served as internal controls. The percentage of specific cell lysis was measured 20 hours later using FACS (R1-gated). Range of percentages of specific lysis is shown for each experimental group. (**D**) Percentages of specific lysis are shown for each experimental group. Data are expressed as the means ± SD of five mice per group. (**E**) Numbers of CD8^+^IFNγ-secreting cells in the spleens of immunized mice were determined by ELISpot using the H-2K^b^-restricted VNHRFTLV peptide. Data are expressed as the means ± SD of five mice per group. ^ΨΨΨ^ *P* < 0.001, rAdVax-immunized compared with saline-injected *T. cruzi*-infected mice. ^§§§^ *P* <0.001, rAdVax-immunized compared with rAdCtrl-injected *T. cruzi*-infected mice.
(TIF)

S2 Fig Heart parasitism and tissue injury are reduced in rAdVax-vaccinated mice challenged with the CL-Brener strain. (**A**) Mice were primed-boosted (s.c.) with 2 × 10^8^ plaque-forming units (PFU) of rAdCtrl or a mixture of 10^8^ PFU of each recombinant adenovirus vaccine construction (rAdASP2+rAdTS; rAdVax) or the vehicle control saline at 6-week intervals. Four weeks after the last immunization, the mice were challenged (i.p.) with 1000 blood trypomastigotes (bt) of the CL-Brener *T. cruzi* Type VI strain and analyzed during the acute phase of infection (23 dpi). (**B**) Quantitative immunohistochemical staining data for *T. cruzi* parasitism (nests/100 microscopic fields) in the heart tissue. (**C**) Evaluation of CK-MB activity in the serum of NI controls and *T. cruzi*-infected mice. The data are presented as the means ± SD of seven to ten mice per group. ***P <0.001, experimental groups compared with NI controls. ^ΨΨ^ *P* < 0.01 and ^ΨΨΨ^ *P* < 0.001 rAdVax-immunized compared with saline-injected *T. cruzi*-infected mice. ^§^ *P* < 0.05 and ^§§^ *P* < 0.01, rAdVax-immunized compared with rAdCtrl-injected *T. cruzi*-infected mice.
(TIF)

S3 Fig rAdVax reduced the number of CD8^+^ cells infiltrating the cardiac tissue in acutely *T. cruzi*-infected mice. Quantification of inflammatory CD4^+^, CD8^+^ and F4/80^+^ (macrophage) cells infiltrating the heart tissue of noninfected (NI) controls and mice injected with saline, rAdCtrl or rAdVax challenged with the Colombian *T. cruzi* Type I strain and analyzed at 50 dpi. The data are presented as the means ± SD of seven to ten mice per group. * *P* <0.05, ** *P* <0.01 and ***P <0.001, experimental groups compared with NI controls. ^ΨΨ^ *P* <0.01, rAdCtrl and rAdVax-immunized compared with saline-injected *T. cruzi*-infected mice. ^§§^ *P* <0.01, rAdVax-immunized compared with rAdCtrl-injected *T. cruzi*-infected mice.
(TIF)

S4 Fig Cytokine expression in the serum of chronically *T. cruzi*-infected mice pre-therapy and pos-therapy with rAdVax. Chronically Colombian-infected mice were evaluated for cytokine expression in the serum at 120 dpi (pre-therapy) or primed-boosted with a mixture of 10^8^ PFU of each adenovirus (rAdASP2+rAdTS) vaccine preparation and analyzed at 230 dpi (pos-therapy). Representative dot plots showing the expression of (**A**) IL-17, IL-4, GM-CSF, TNF and IFNγ or (**B**) IL-10, IL-6, IL-5, IL-2 and IL-1α.
(TIF)

S5 Fig NO_x_ levels in the serum are associated with cardiomyocyte lesion in chronic *T. cruzi* infection. (**A**) Concentrations of NO_x_ in the serum of noninfected (NI) controls and Colombian-infected C57BL/6 mice. (**B**) No correlation was observed between parasitemia and CK-MB activity levels during the chronic phase of infection. The results represent five to twelve mice per experimental group. * *P* <0.05, ** *P* <0.01 and ***P <0.001, experimental groups compared with NI controls.
(TIF)
