# Supplementary figures and images for: A Human Type 5 Adenovirus-Based Trypanosoma cruzi Therapeutic Vaccine Re-programs Immune Response and Reverses Chronic Cardiomyopathy
Source: PLoS Pathog. 2015 Jan 24;11(1):e1004594. doi: 10.1371/journal.ppat.1004594 (PMC4305326; doi:10.1371/journal.ppat.1004594)

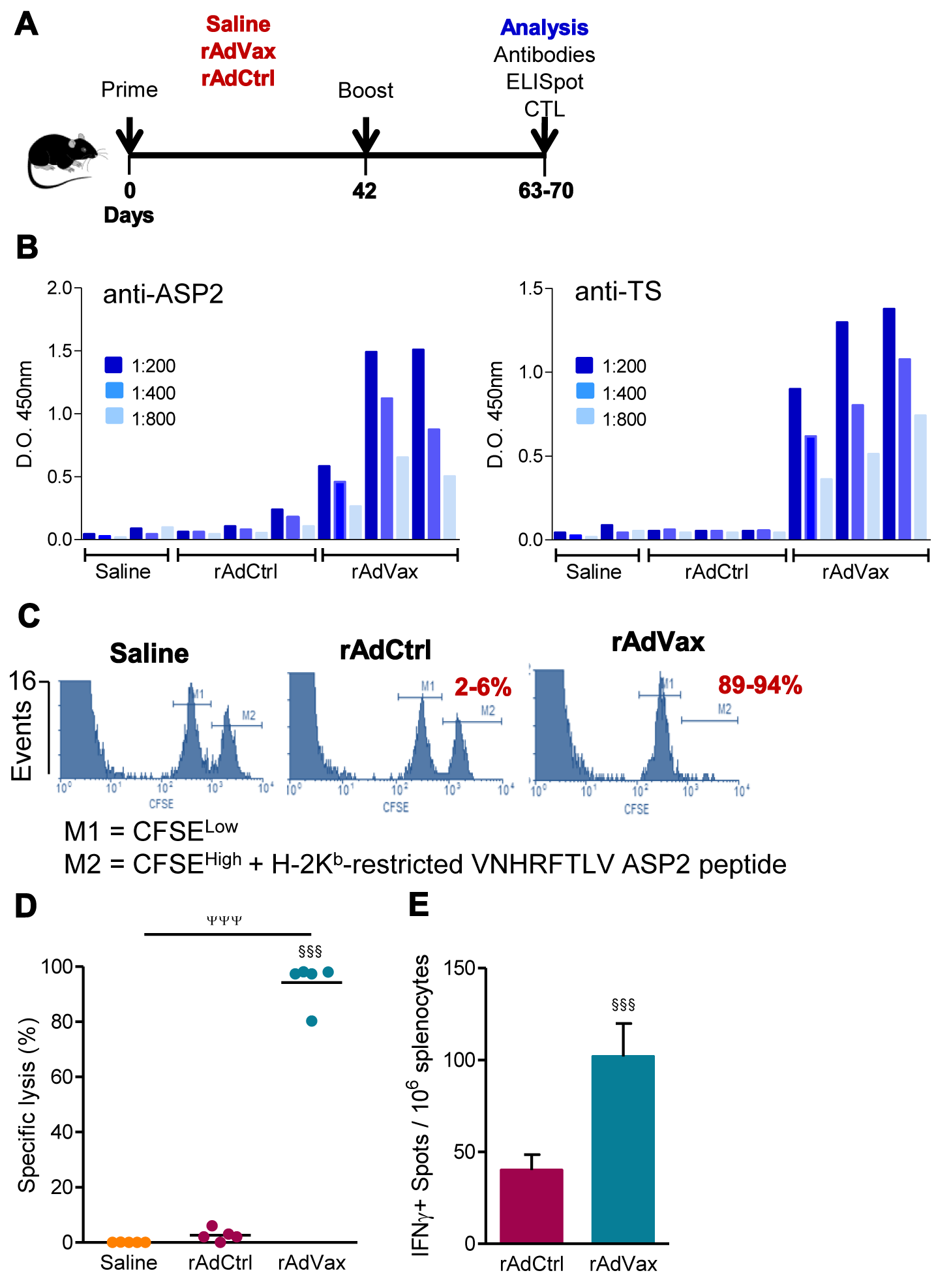

Supplement: S1 Fig — (A) Mice were s.c. primed-boosted with 2 × 10^8^ plaque-forming units (PFU) of rAdCtrl or a mixture of 108 PFU of each adenovirus vaccine preparation (rAdASP2+rAdTS; rAdVax) or the vehicle control saline at 6-week intervals. (B) Total (IgM+IgG) antibodies to ASP2 and TS peptides were detected using ELISA. Each serum sample was serially diluted (1:200, 1:400 and 1:800) before analysis. The data are presented as the means of three to five mice serum samples per group. (C) Representative histogram of in vivo cytotoxicity assays of normal splenocytes labeled with two concentrations of CFSE (CFSEHigh and CFSELow) that were inoculated intravenously into the tail vein of immunized mice. CFSEHigh cells were pulsed with the ASP2 peptide. CFSELow cells were unpulsed and served as internal controls. The percentage of specific cell lysis was measured 20 hours later using FACS (R1-gated). Range of percentages of specific lysis is shown for each experimental group. (D) Percentages of specific lysis are shown for each experimental group. Data are expressed as the means ± SD of five mice per group. (E) Numbers of CD8+IFNγ-secreting cells in the spleens of immunized mice were determined by ELISpot using the H-2Kb-restricted VNHRFTLV peptide. Data are expressed as the means ± SD of five mice per group. ΨΨΨ P < 0.001, rAdVax-immunized compared with saline-injected T. cruzi-infected mice. §§§ P <0.001, rAdVax-immunized compared with rAdCtrl-injected T. cruzi-infected mice. (TIF) [file ppat.1004594.s001.tif]

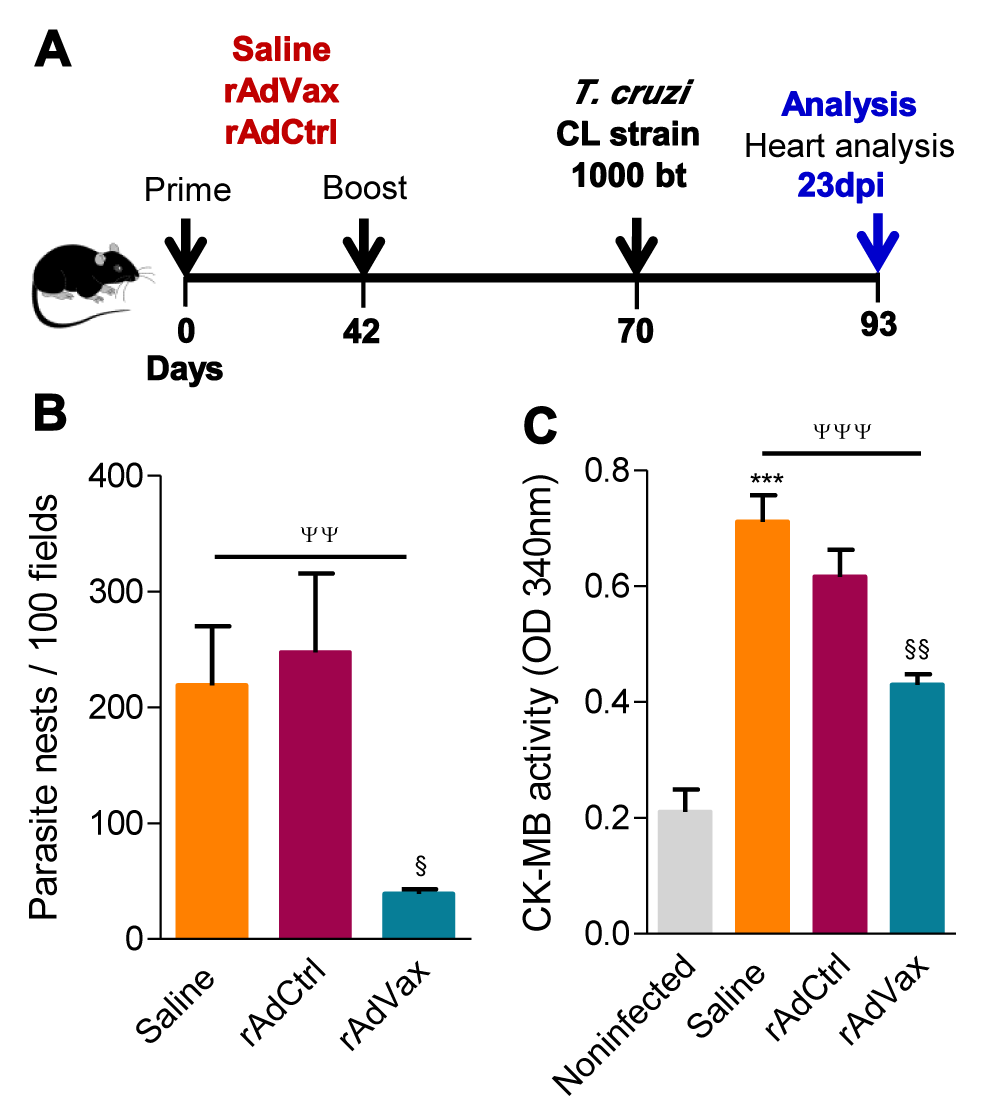

Supplement: S2 Fig — (A) Mice were primed-boosted (s.c.) with 2 × 10^8^ plaque-forming units (PFU) of rAdCtrl or a mixture of 108 PFU of each recombinant adenovirus vaccine construction (rAdASP2+rAdTS; rAdVax) or the vehicle control saline at 6-week intervals. Four weeks after the last immunization, the mice were challenged (i.p.) with 1000 blood trypomastigotes (bt) of the CL-Brener T. cruzi Type VI strain and analyzed during the acute phase of infection (23 dpi). (B) Quantitative immunohistochemical staining data for T. cruzi parasitism (nests/100 microscopic fields) in the heart tissue. (C) Evaluation of CK-MB activity in the serum of NI controls and T. cruzi-infected mice. The data are presented as the means ± SD of seven to ten mice per group. ***P <0.001, experimental groups compared with NI controls. ΨΨ P < 0.01 and ΨΨΨ P < 0.001 rAdVax-immunized compared with saline-injected T. cruzi-infected mice. § P < 0.05 and §§ P < 0.01, rAdVax-immunized compared with rAdCtrl-injected T. cruzi-infected mice. (TIF) [file ppat.1004594.s002.tif]

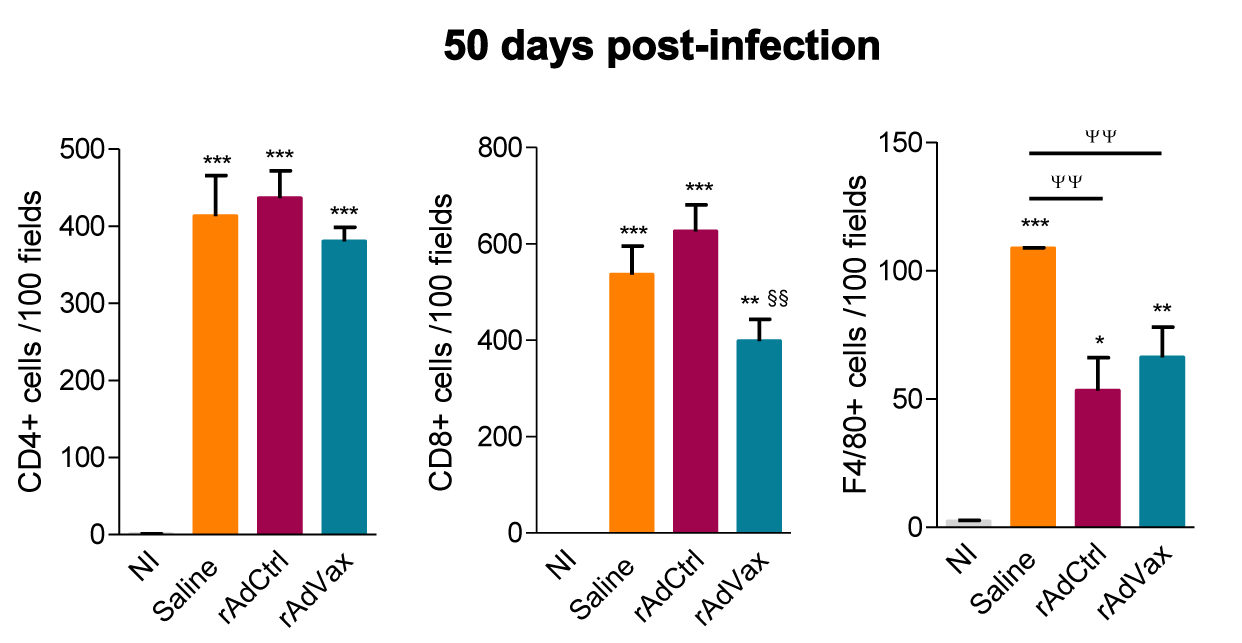

Supplement: S3 Fig — Quantification of inflammatory CD4+, CD8+ and F4/80+ (macrophage) cells infiltrating the heart tissue of noninfected (NI) controls and mice injected with saline, rAdCtrl or rAdVax challenged with the Colombian T. cruzi Type I strain and analyzed at 50 dpi. The data are presented as the means ± SD of seven to ten mice per group. * P <0.05, ** P <0.01 and ***P <0.001, experimental groups compared with NI controls. ΨΨ P <0.01, rAdCtrl and rAdVax-immunized compared with saline-injected T. cruzi-infected mice. §§ P <0.01, rAdVax-immunized compared with rAdCtrl-injected T. cruzi-infected mice. (TIF) [file ppat.1004594.s003.tif]

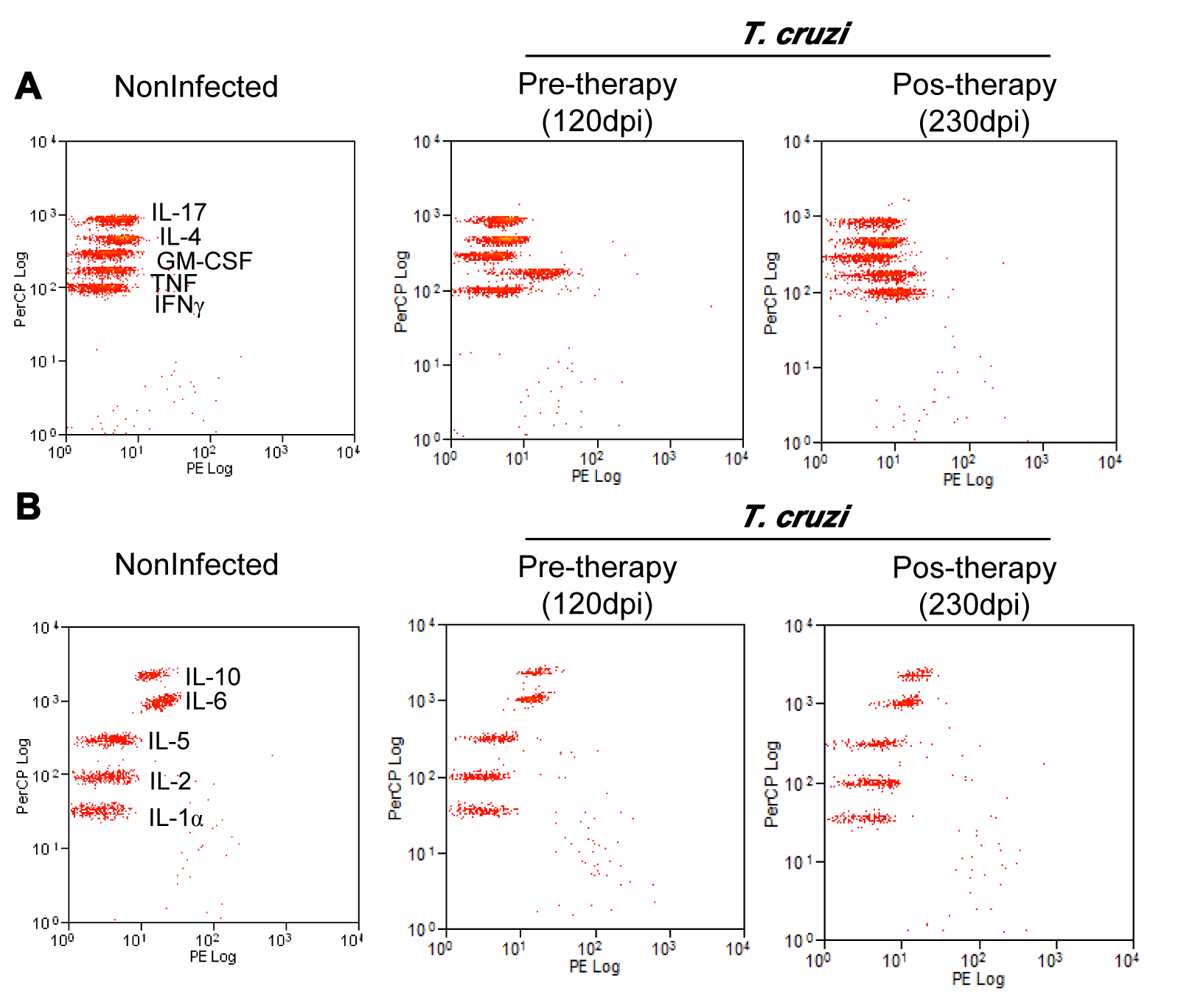

Supplement: S4 Fig — Chronically Colombian-infected mice were evaluated for cytokine expression in the serum at 120 dpi (pre-therapy) or primed-boosted with a mixture of 108 PFU of each adenovirus (rAdASP2+rAdTS) vaccine preparation and analyzed at 230 dpi (pos-therapy). Representative dot plots showing the expression of (A) IL-17, IL-4, GM-CSF, TNF and IFNγ or (B) IL-10, IL-6, IL-5, IL-2 and IL-1α. (TIF) [file ppat.1004594.s004.tif]

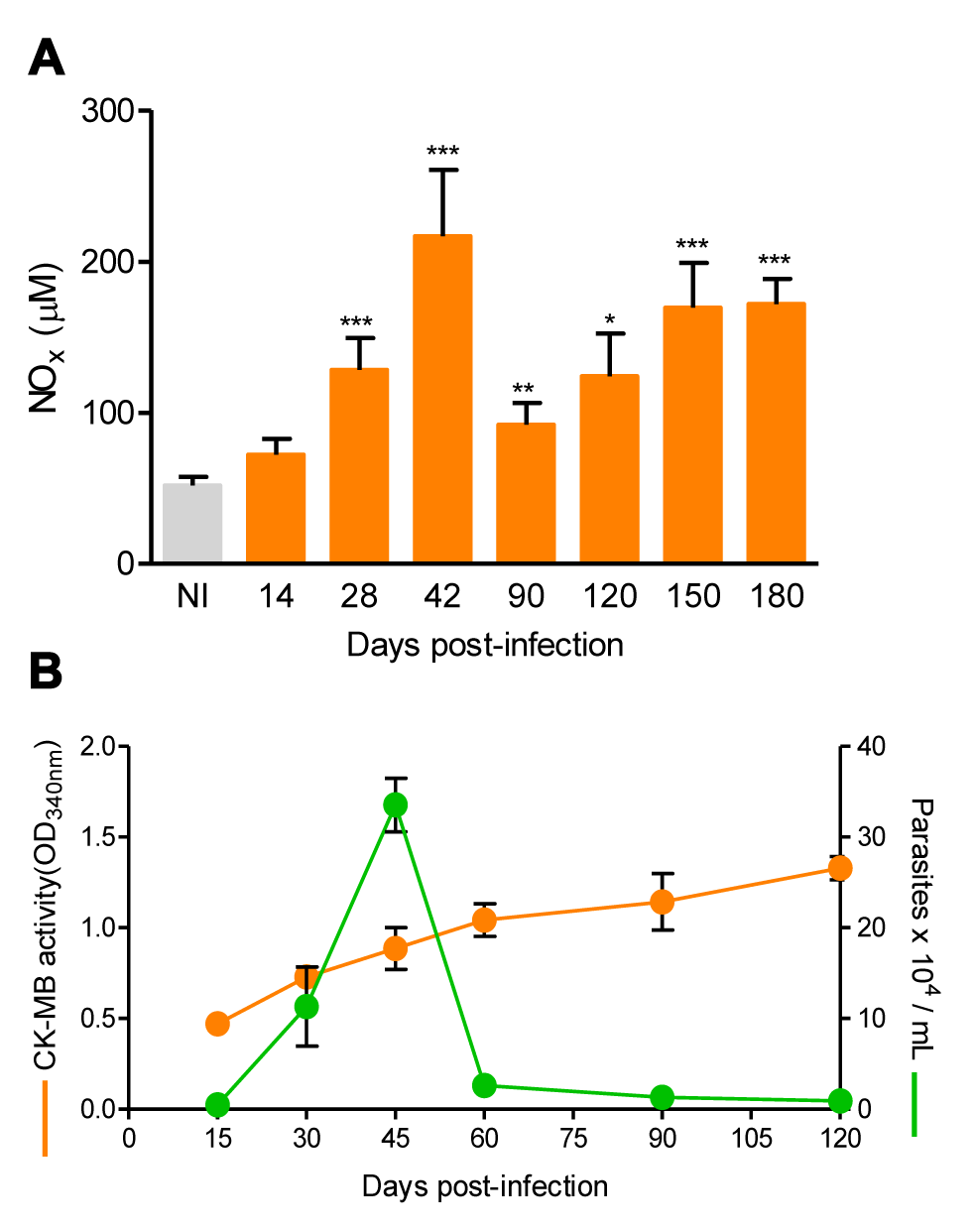

Supplement: S5 Fig — (A) Concentrations of NOx in the serum of noninfected (NI) controls and Colombian-infected C57BL/6 mice. (B) No correlation was observed between parasitemia and CK-MB activity levels during the chronic phase of infection. The results represent five to twelve mice per experimental group. * P <0.05, ** P <0.01 and ***P <0.001, experimental groups compared with NI controls. (TIF) [file ppat.1004594.s005.tif]
